# Diverse Structural Design Strategies of MXene-Based Macrostructure for High-Performance Electromagnetic Interference Shielding

**DOI:** 10.1007/s40820-023-01203-5

**Published:** 2023-11-02

**Authors:** Yue Liu, Yadi Wang, Na Wu, Mingrui Han, Wei Liu, Jiurong Liu, Zhihui Zeng

**Affiliations:** 1 Key Laboratory for Liquid-Solid Structural Evolution and Processing of Materials, School of Materials Science and Engineering, Shandong University, Jinan, 250061 People’s Republic of China; 2 Department of Mechanical Engineering, The Hong Kong Polytechnic University, Kowloon, Hong Kong 999077 People’s Republic of China; 3 State Key Laboratory of Crystal Materials, Institute of Crystal Materials, Shandong, 250100 China; 4 Shenzhen Research Institute of Shandong University, Shenzhen, China; 5 School of Chemistry and Chemical Engineering, Shandong University, Shandong, 250100 China

**Keywords:** MXene, Composite, Electromagnetic interference shielding, Microstructure, Electronics

## Abstract

There is an urgent demand for flexible, lightweight, mechanically robust, excellent electromagnetic interference (EMI) shielding materials. Two-dimensional (2D) transition metal carbides/nitrides (MXenes) have been potential candidates for the construction of excellent EMI shielding materials due to their great electrical electroconductibility, favorable mechanical nature such as flexibility, large aspect ratios, and simple processability in aqueous media. The applicability of MXenes for EMI shielding has been intensively explored; thus, reviewing the relevant research is beneficial for advancing the design of high-performance MXene-based EMI shields. Herein, recent progress in MXene-based macrostructure development is reviewed, including the associated EMI shielding mechanisms. In particular, various structural design strategies for MXene-based EMI shielding materials are highlighted and explored. In the end, the difficulties and views for the future growth of MXene-based EMI shields are proposed. This review aims to drive the growth of high-performance MXene-based EMI shielding macrostructures on basis of rational structural design and the future high-efficiency utilization of MXene.

## Highlights

- MXene-based macrostructure development and EMI shielding mechanisms are reviewed.
- Various structural design strategies for MXene-based EMI shielding materials are highlighted and discussed.
- Current challenges and future directions for MXenes in electromagnetic interference shielding are outlined.

## Graphical abstract

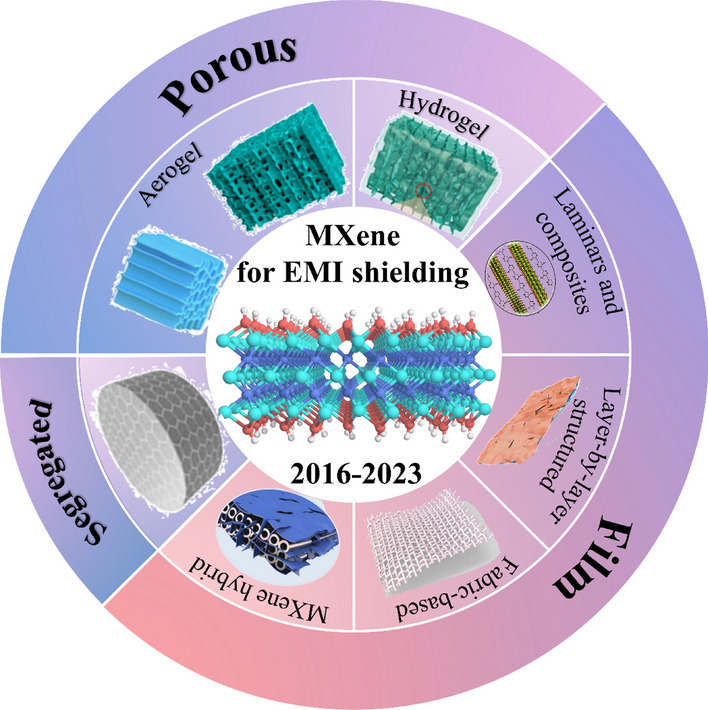

## Introduction

The complications associated with electromagnetic interference (EMI), which is caused by adverse electromagnetic waves (EMWs) or radiation, are becoming more severe as high-tech industries continue to expand. EMI adversely affects electrical devices and equipment, interferes with their functioning, contaminates the environment, and jeopardizes human health [1–3]. EMI shielding inhibits or attenuates EMWs transmission between the protected region and surrounding environment. In theory, EMWs are reflected and absorbed by shielding materials [4–7]. Therefore, the purpose of EMI shields is to eliminate EMI, which has significant implications for the development of next-generation electronics, communication technology, Internet of Things, and aerospace technology. Although conventional metal materials, characterized by great electrical conductivity, perform well as EMI shields, their inherently grreat density, bad corrosion stability, and demanding processability severely restrict their usage in greatly integrated modern mobile electronics [8–10]. Thus, composites containing various fillers [11], magnetic fillers [12], and dielectric fillers, have been created as substitutes for metals in EMI shielding applications [13–16]. Although these composites are lightweight and have anticorrosive nature, their restricted shielding capacities and complex processability restrict their widespread utilization [17].

**Fig. 1:**
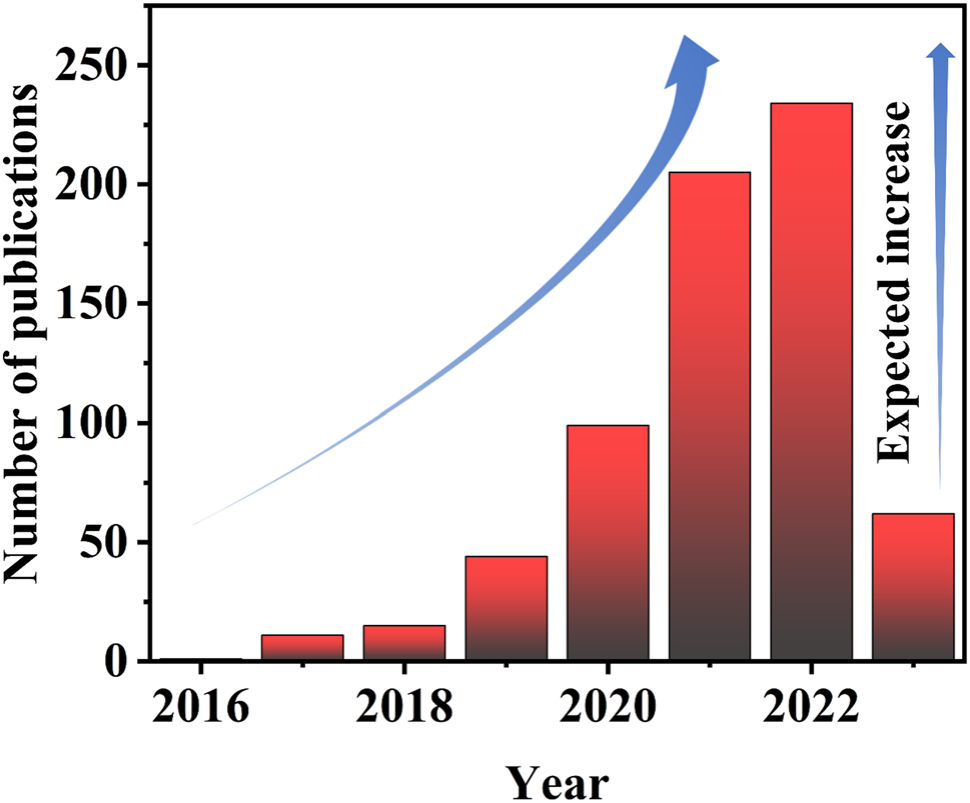
The number of studies concerning "MXene" and "Electromagnetic shielding" published from 2016 to 2023. Source: Web of Science (Search time: March 2023)

MXenes are 2D transition metal carbides and/or nitrides with the generic formula *M*_*n*+1_*X*_*n*_*T*_*x*_ (*n* = 1–3) [18–20]. The selective etching of a layer, typically comprising a group IIIA or group IVA factor, is often performed to match the MAX phase precursors (*M*_*n*+1_*AX*_*n*_) and create layered MXene materials. The distinctive 2D layered structure and rich surface functional groups give MXenes remarkable features, including high conductivity, surface hydrophilicity, and mechanical steadiness [21]. Owing to their exceptional qualities, MXenes can be adopted in a broad range of usage, including supercapacitors, alkali metal ion storage devices and sensor platforms. Moreover, MXenes show significant potential for the growth of EMI shielding materials because they possess the necessary fundamental properties for constructing effective EMI shielding macrostructures, including excellent mechanical properties, remarkable electrical conductivity, big given surface areas and aspect ratios, and, most importantly, simple processability in aqueous media [22–25]. Because the first report on the exceptional EMI shielding capabilities of Ti_3_C_2_T_*x*_ MXenes in 2016, research in this field has proliferated exponentially (Fig. 1). Therefore, reviews centered on the development and future of EMI shielding materials on basis of MXene are highly desirable. Although there are some reviews of electromagnetic shielding materials based on MXene, these articles mainly explore MXene components, rather than the structure of MXene-based electromagnetic shielding composites. This review pays attention to the progress in the assembly of MXene-based EMI shielding macrostructures, as well as the associated EMI shielding mechanisms. Meanwhile, diverse structural design strategies for MXene-based EMI shielding materials are emphasized on basis of their inherent properties. In the end, the difficulties and views accompanying the development of MXene-based EMI shields are discussed. We hope that this material will serve as guidance for developing high-performance MXene-based EMI shielding macrostructures on basis of the rational design of components and structures and contribute to the high-efficiency utilization of emerging MXene nanomaterials.

## EMI Shielding Mechanisms

**Fig. 2:**
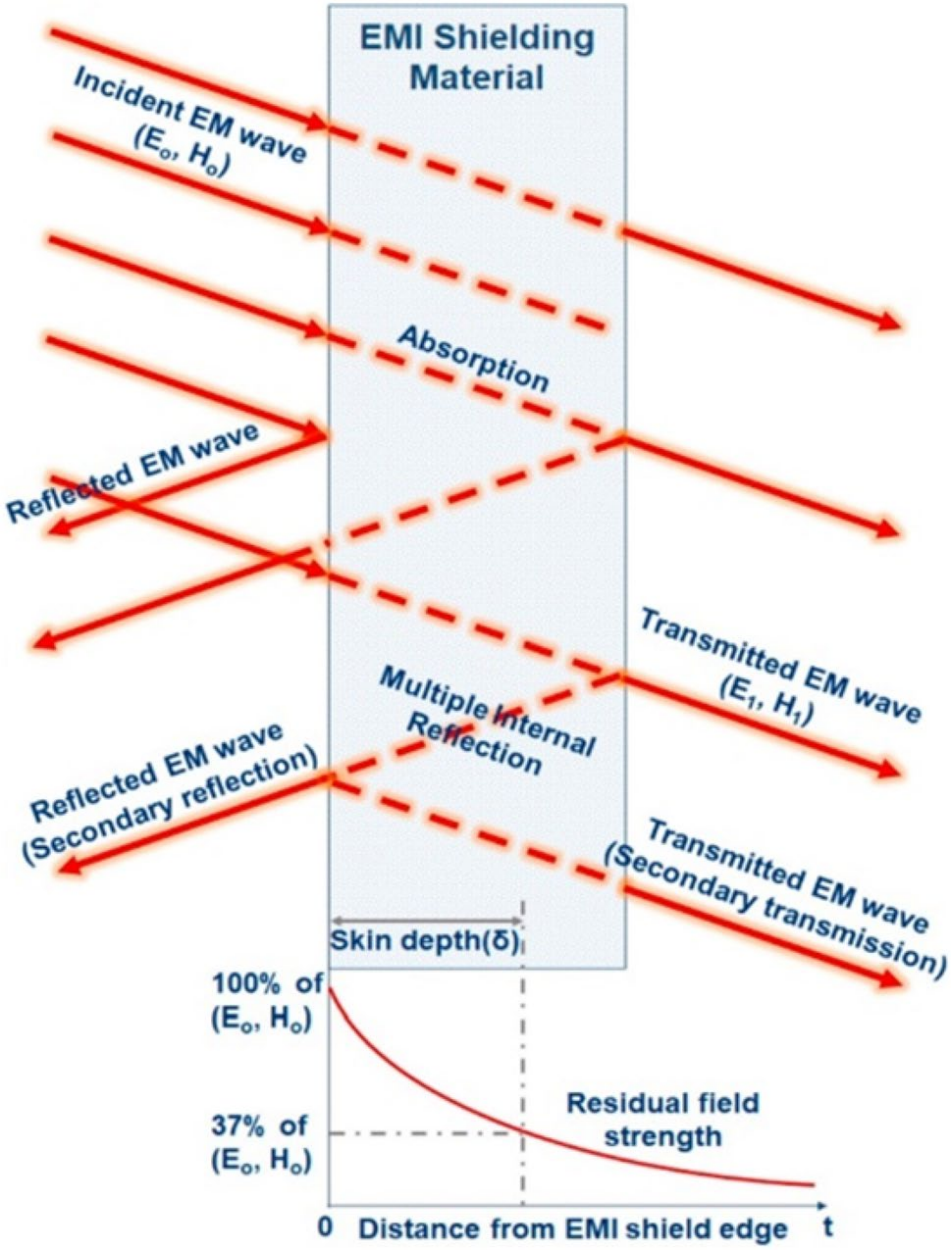
Graphical representation of EMI shielding mechanisms. Reproduced with consent from Ref. [31]. Copyright 2018, Elsevier

Electromagnetic shielding is an important strategy for suppressing EMI and protecting against electromagnetic radiation. Electromagnetic shielding means the application of conductive or magnetically permeable materials as shields to block the propagation EMWs and weaken the electromagnetic field [26]. For understanding EMI shielding intrinsically, different shielding systems have been put forward, including the eddying role, electromagnetic field, and transmission line theories. The transmission line theory has been extensively implemented owing to the advantages of simplicity, and convenient and accurate calculation. The transmission line theory is often used to illustrate electromagnetic shielding mechanisms [27–29] that comprise three distinct processes, namely, reflection, absorption, and multiple reflection [30]. Specifically, incident EMWs reaching the surface of a shielding material are partially shown as a result of the mismatch between the shielding material and air. The non-shown EMWs enter the shielding material where they are reflected numerous times and absorbed. The remaining small part of EMWs pass by the shielding material as transmitted waves. Figure 2 describes the EMI shielding system [31].

### EMI Shielding Performance

The degree of incident EMWs attenuation is a key indicator for evaluating the shielding ability of a material. EMI shielding efficacy (EMI SE, unit: dB) is employed for quantitatively expressing the shielding capacity of a material [32]. It is represented by the proportion of the electric field strength, magnetic field strength, or effect of EMWs before and after they pass by the shielding material, as shown in Eq. (1) [33, 34]:

1 $${\text{SE}} = 20\log_{10} \left( {\frac{{E_{t} }}{{E_{i} }}} \right) = 20\log_{10} \left( {\frac{{H_{t} }}{{H_{i} }}} \right) = 20\log_{10} \left( {\frac{{P_{t} }}{{P_{i} }}} \right)$$

where the subscripts i and t show incident and transmitted EMWs.

Based on the Schelkunoff theory [35, 36], the overall EMI SE is the total reflections (SE_*R*_), absorptions (SE_*A*_), and multiple reflections (SE_*M*_) of EMWs by a shielding material [37–39].

2 $${\text{SE}}_{T} = {\text{SE}}_{R} + {\text{SE}}_{A} + {\text{SE}}_{M}$$

#### Reflection Loss (SE_*R*_)

SE_*R*_ is brought by an impedance mismatch between the propagation medium of the incident EMWs and the surface of the shielding material. The SE_*R*_ magnitude from the front to the back surface of the shield can be shown below [40]:

3 $${\text{SE}}_{R} = 20\log_{10} \left( {\frac{{Z + Z_{0} }}{{4ZZ_{0} }}} \right) = 39.5 + 10\log_{10} \frac{\sigma }{2f\pi \mu }$$

where *Z* and *Z*_0_ impede the shield material and air; $\sigma$ and $\mu$ stand for the electrical electroconductibility and the magnetic penetrability; and $f$ stands for the frequency of the incident EMWs. According to the formula, *SE*_R_ is associated with the electroconductibility and magnetic penetrability of the material and the frequency of the EMWs.

#### Absorption Loss (SEA)

SE_A_ is mainly derived from the dielectric and magnetic losses of a material [41]. To achieve great absorption loss, the conditions below are required: high electrical conductivity for ohmic loss, which increases interactions between the electrons and incident EMWs [42]; high dielectric and magnetic permeabilities increase the eddy current loss and magnetic hysteresis loss [43]. The absorbed electromagnetic energy is dissipated as heat. The absorption loss (SE_*A*_) of a shielding material can be shown as follows [44–46]:

4 $${\text{SE}}_{A} = 20\left( {\frac{d}{\delta }} \right)\log_{10} e = 8.68\left( {\frac{d}{\delta }} \right) = 8.68d\frac{{\sqrt {f\mu \sigma } }}{2}$$

where *d* means the thickness of the shielding material, and $\delta$ stands for the skin or penetration depth. For conductive shields, the skin depth is shown as $\delta = \frac{1}{\alpha } = \left( {\sqrt {\pi f\sigma \mu } } \right)^{ - 1}$. Here, $\alpha$ means the EMWs attenuation constant of the shielding material and is expressed as $\alpha = \omega \sqrt {\frac{\mu \varepsilon }{2}\left[ {\sqrt {1 + \left( {\frac{\sigma }{\omega \varepsilon }} \right)^{2} } - 1} \right]}$, where $\omega$ stands for the angular frequency ($2\pi f$) and $\varepsilon$ is the dielectric permittivity. Hence, SE_A_ depends on the controllable thickness and electrical conductivity and on the inherent electromagnetic nature of the material.

#### Multiple Reflections (SEM)

Various reflections of EMWs between two boundaries of a thin shield result in their attenuation. This process can be repeated until the electromagnetic energy dissipates completely. Multireflection effectiveness is calculated as follows [47, 48]:

5 $${\text{SE}}_{M} = 20\log_{10} \left( {1 - e^{{\frac{ - 2d}{\delta }}} } \right) = 20\log_{10} \left( {1 - 10^{{\frac{{SE_{A} }}{10}}} } \right)$$

SE_*M*_ is majorly dependent on the thickness of the material. This parameter can be neglected when the shield is thicker than the skin depth, or in case of the SE_*T*_ of above 15 dB [50, 51]. Nevertheless, if the thickness is significantly thinner than the skin depth, various reflections must be regarded while evaluating shield efficacy.

#### Internal Scattering (Internal Multiple Reflections)

In addition to the macroscopic reflections occurring between the two boundaries of an EMI shield, reflections and scattering inside the microstructure of the EMI shielding material can also occur. Such internal scattering (internal multiple reflections) may additionally prolong the propagation pathway of EMWs, thereby enhancing absorption loss and the total shielding effect. Therefore, the microstructures comprising a shielding material exert an important effect on EMI shielding.

#### Calculation of EMI Shielding Performance

For practical calculating EMI SE, the calculation theory on basis of either absorption or reflection loss is favored, depending on which property can be directly calculated from the scattering coefficients (*S*-parameters). Here, absorption loss and reflection loss are represented by SE_*A*'_ and SE_*R*'_, respectively, to distinguish them from the concepts with the same name in the Schelkunoff theory:

6 $${\text{SE}}_{R^{\prime}} = 10\log_{10} \left| {\frac{1}{{1 - S_{11}^{2} }}} \right|$$

7 $${\text{SE}}_{A^{\prime}} = 10\log_{10} \left| {\frac{{1 - S_{11}^{2} }}{{S_{21}^{2} }}} \right|$$

It is worth noting that macroscopic multiple reflection losses are encompassed by SE_*A*'_ and SE_*R*'_; thus, SE is defined as follows [49]:

8 $${\text{SE}} = {\text{SE}}_{R^{\prime}} + {\text{SE}}_{A^{\prime}}$$

In addition, taking into account the density and thickness of the material, the feasible shielding effect of a material was defined by introducing the absolute shielding efficiency (SSE/*t*), which is calculated below:

9 $$\frac{{{\text{SSE}}}}{t} = \frac{{{\text{EMI}}\;{\text{SE}}}}{\rho t} = {\text{dB}}\;{\text{cm}}^{2} {\text{g}}^{ - 1}$$

where $\rho$ and $t$ denote the density and thickness of the shielding material, respectively. A great SSE/*t* proportion is required for lightweight shielding usage.

### EMI Shielding Mechanism of MXenes

MXenes are metal carbide or nitride materials characterized by a 2D layered construction, great electrical conductivity, big given surface area, and rich functional groups [52]. The favorable EMI shielding nature of these materials can be partly attributed to their great electrical electroconductibility [53] (10,000 S cm^−1^) and layered structure. When incident EMWs reach the MXene surface, a portion is shown because of the rich free electrons on the surface of the greatly conductive MXene [54]. The EMWs enter the MXene lattice construction and make interaction with the dense electron to generate an electric current, resulting in ohmic losses, which attenuate EMWs. Moreover, layered structures can effectively enhance internal multiple scattering and reflection of EMWs; therefore, lamellar MXenes have been proven to be superior internal multiple reflection filler materials for EMI shielding [55].

## MXenes as EMI Shielding Materials

### MXene-Based Porous Materials

Lightweight design is becoming increasingly important in EMI shielding applications [44]. In addition to effectively reducing the weight of composite materials, porous structures also promote multiple reflections of EMWs inside the material, thereby enhancing EMWs absorption and improving the shielding effect.

#### Foams and Aerogels

MXenes generally agglomerate readily, which limits the establishment of an effective conductive network. The introduction of porous structures can decrease the MXene agglomeration and improve the overall conductivity and electromagnetic shielding capacity of the material. Additionally, the weak interactions between MXene nanosheets preclude the construction of a three-dimensional structure with excellent mechanical properties using MXene alone; therefore, it is necessary to prepare MXene-based composites. MXene-based porous foams and aerogels have been widely studied as they are light-weight, highly porous, low-density, and adequately conductive.

**Fig. 3:**
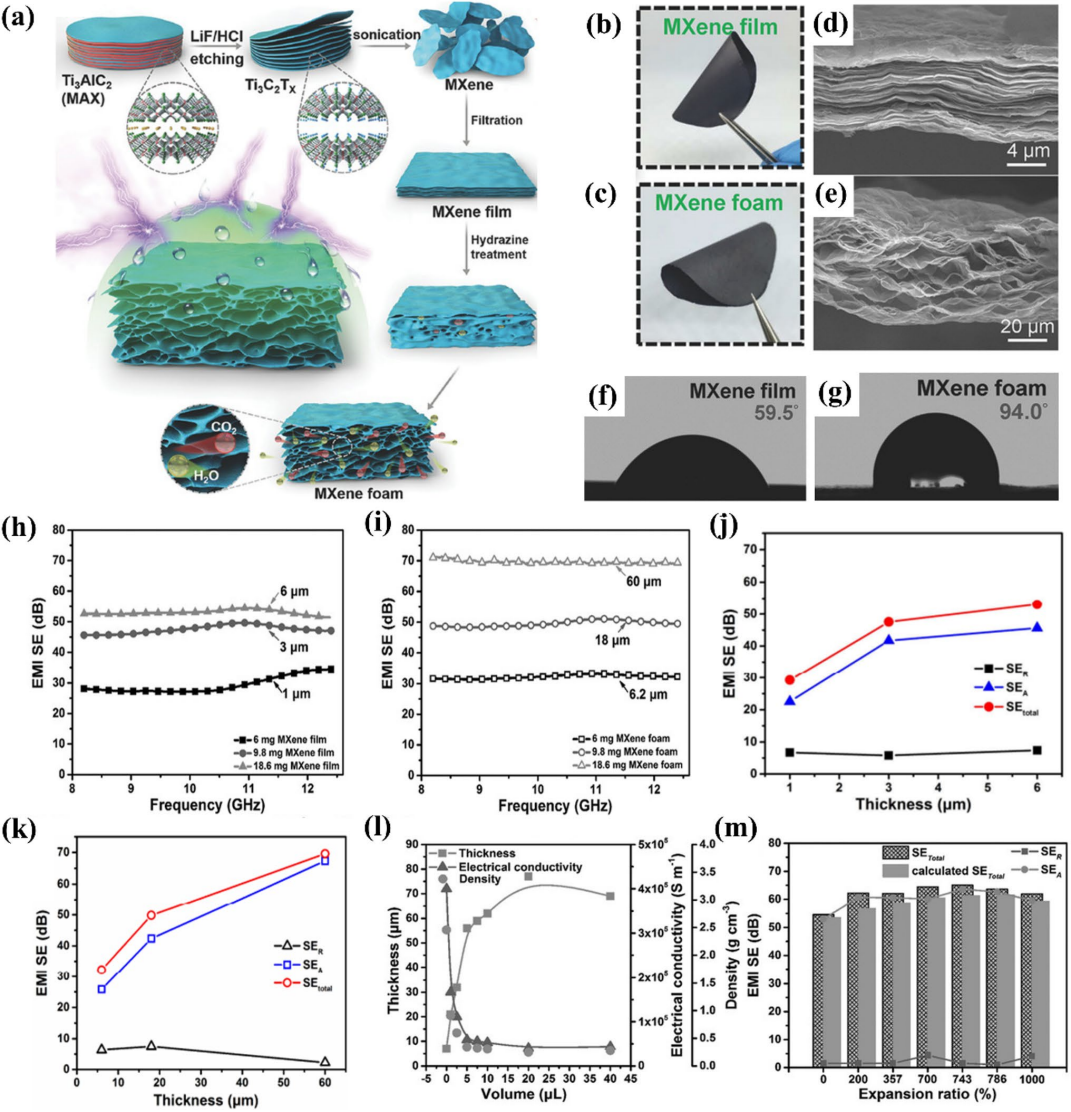
**a** Graphical illustration showing the generation of MXene foams; digital figures of the MXene, **b** film, and **c** foam. Cross-sectional scanning electron microscopy images of the MXene **d** film and **e** foam. Contact angle measurements of the MXene **f** film and **g** foam. EMI SEs of MXene **h** films and **i** foams of different thicknesses. Contrast of the mean SE_T_, SE_R_, and SE_A_ of the MXene **j** films and **k** foams of varying thicknesses over the X-band. **l** Thicknesses, densities, and electrical conductivities of the MXene foams using varying doses of hydrazine. **m** Roles of the expansion proportion in the EMI SEs of the MXene foams. Reproduced with consent from Ref. [56]. Copyright 2017, WILEY–VCH

Liu et al. produced freestanding, flexible, reasonably strong, and hydrophobic MXene foams through fitting MXene sheets into thin films [56]. By comparing with famous hydrophilic MXene materials, the MXene foam exhibited a startlingly hydrophobic surface, and excellent water resistance and durability. The MXene film was 6 μm thick, and its EMI SE was 53 dB, whereas that of the MXene porous foam grew to 60 μm and its EMI SE grew to 70 dB (Fig. 3a–g). The porous structure promoted EMWs scattering and reflection, effectively dissipating the EMWs energy. Notably, the EMI shielding system of the foam and film was absorption loss, which greatly reduced secondary electromagnetic pollution (Fig. 3h–k). Increasing the hydrazine dose resulted in increased thickness of the material and reduced density and conductivity. However, because the MXene foam formed a solid conductive network, the shielding properties of the material did not change significantly (Fig. 3l). The MXene foam showed comparable, high SE values and dependence on the expansion rate in both the experimental and calculated outcomes (Fig. 3m).

**Fig. 4:**
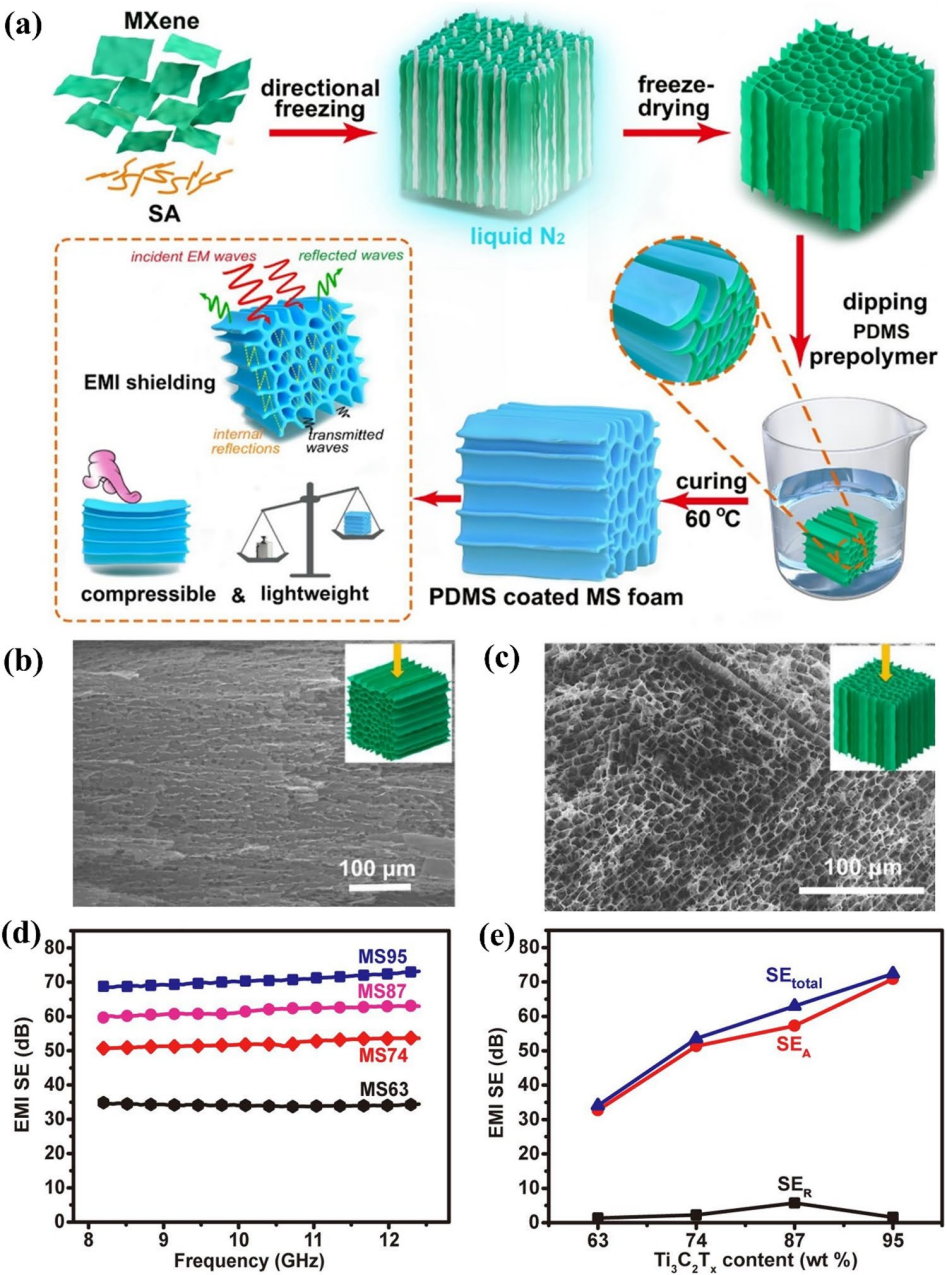
**a** Graphical description depicting the generation of MXene/SA hybrid aerogels and PDMS-coated MS foam. SEM figures of MS74 (mass proportion of MXene was 74%), **b** aerogel side figure and **c** top figure. **d** EMI SEs of the MS aerogels, and **e** plots of EMI SEs of the MS aerogels vs. the MXene content. Reproduced with consent from Ref. [57]. Copyright 2020, Elsevier

Using highly thermally conductive copper plates, Wu et al. constructed unidirectional aerogels coated with polydimethylsiloxane (PDMS) via directional freezing of MXene/sodium alginate (SA). These lightweight, compressible aerogels with a honeycomb-like structure showed excellent conductivities of up to 2211 S m^−1^ and impressive EMI SEs of above 72 dB (Fig. 4) [57]. The MXene/SA aerogels exhibited high conductivities of up to 644 S m^−1^ and great EMI SEs. The PDMS coating imparts considerable compressibility and durability to 3D conductive networks, broadening the potential applicability of composite foams to wearable devices and sensors. The foam coated with 6.1 wt% PDMS maintained the shielding efficiency at 48.2 dB. As MXene content increases, the SE_T_, and SE_A_ increased significantly, while the SE_R_ remained below 5 dB, suggesting that the electromagnetic shielding capacity of the foam was ruled by absorption loss, and that the internal conductive network facilitated EMWs absorption.

**Fig. 5:**
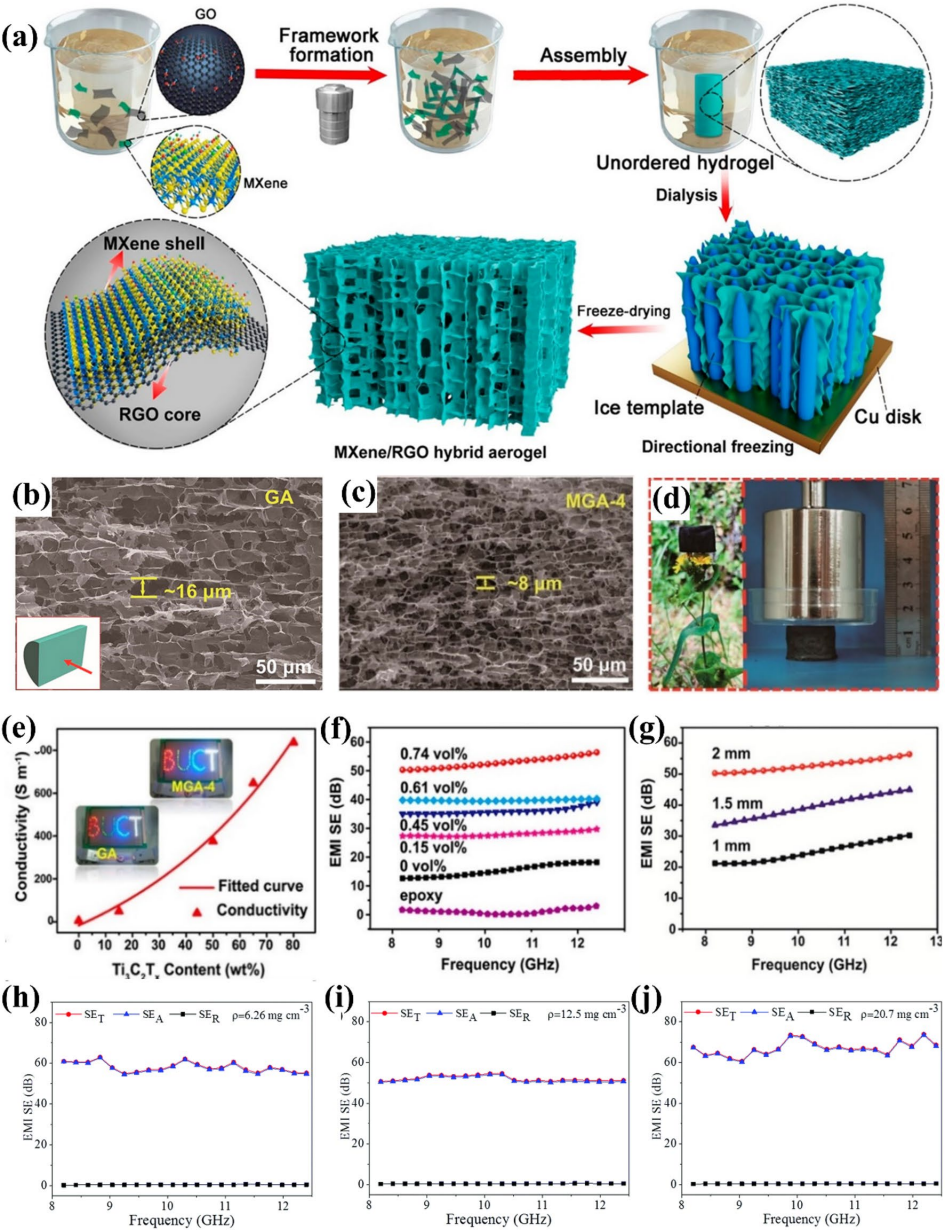
**a** Schematically describing the generation of MXene/RGO hybrid aerogels. **b** SEM figure of GA (graphene aerogel). **c** Side figure SEM image of MGA-4 (MGA-4 contains 80 wt% Ti_3_C_2_T_*x*_). **d** Digital figures of MGA-4 (100 mg) resting on a flower (left) and supporting a weight of 500 g (right). **d** MXene aerogel (35 cm^3^ g^−1^) resting on foxtail grass. **e** Conductivities of the MXene/RGO hybrid aerogels. **f** EMI SE of EP/MGA nanocomposites. **g** Role of sample thickness in the EMI SE of EP/MGA-4 nanocomposite (0.74 vol%). Total, reflection, and absorption EMI SEs of MXene aerogels with varying densities **h** 6.26 mg cm^−3^, **i** 12.5 mg cm^−3^, **j** 20.7 mg cm^−3^. **a**–**c** and **e**–**g** Reproduced with consent from Ref. [58]. Copyright 2018, American Chemical Society. **d** and **h**–**j** Reproduced with consent from Ref. [59]. Copyright 2019, Royal Society of Chemistry

Zhao et al. constructed porous and conductive 3D MXene sheet structures via graphene oxide (GO)-helped hydrothermal assembly (Fig. 5a–c) [58]. The MXene/RGO hybrid aerogel (MGA) presented a uniform cellular microstructure comprising intact MXene and exhibited an ultra-high electroconductibility of 1085 S m^−1^. After compositing with epoxy resin, the composite aerogel displayed a great electroconductibility of 23.3 S m^−1^ and an EMI SE of 29.7 dB. When the fraction of MXene reached 0.74 vol%, the electroconductibility and EMI SE of the composites grew to 695.9 S m^−1^ and 56.4 dB (Fig. 5e–g).

Bian et al. prepared large-sized honeycomb-structured ultralight MXene aerogels comprising freeze-dried single-layered MXene (Fig. 5d) [59]. An MXene dispersion was first frozen, leading to the generation of ice crystals along the MXene sheets; the sheets were repelled by the ice crystal borders to build a steady network of connections. Subsequent freeze-drying sublimated the ice crystals while the MXene sheets maintained their original structure, thereby generating a large number of holes. The MXene aerogels showed great EMI shielding performances. The given shielding effect reached 9904 dB cm^3^ g^−1^ (Fig. 5h–j).

The great conductivity and electromagnetic shielding performances of the above two types of aerogels are largely attributed to their intricate 3D conductive network and porous structure. The size of the internal micropores was determined by the ice crystal volume, and could thus be regulated by adjusting the concentration of MXene in the aqueous dispersion.

#### Hydrogels

Abundant pores within a porous aerogel impede the penetration of EMWs; however, the air inside the pores limits further EMWs attenuation. Hydrogels contain water in their pores, thereby retaining the advantages of a porous structure, while being capable of further attenuating incident EMWs. Hydrogels have received widespread attention because of their favorable biocompatibility and robustness [130].

**Fig. 6:**
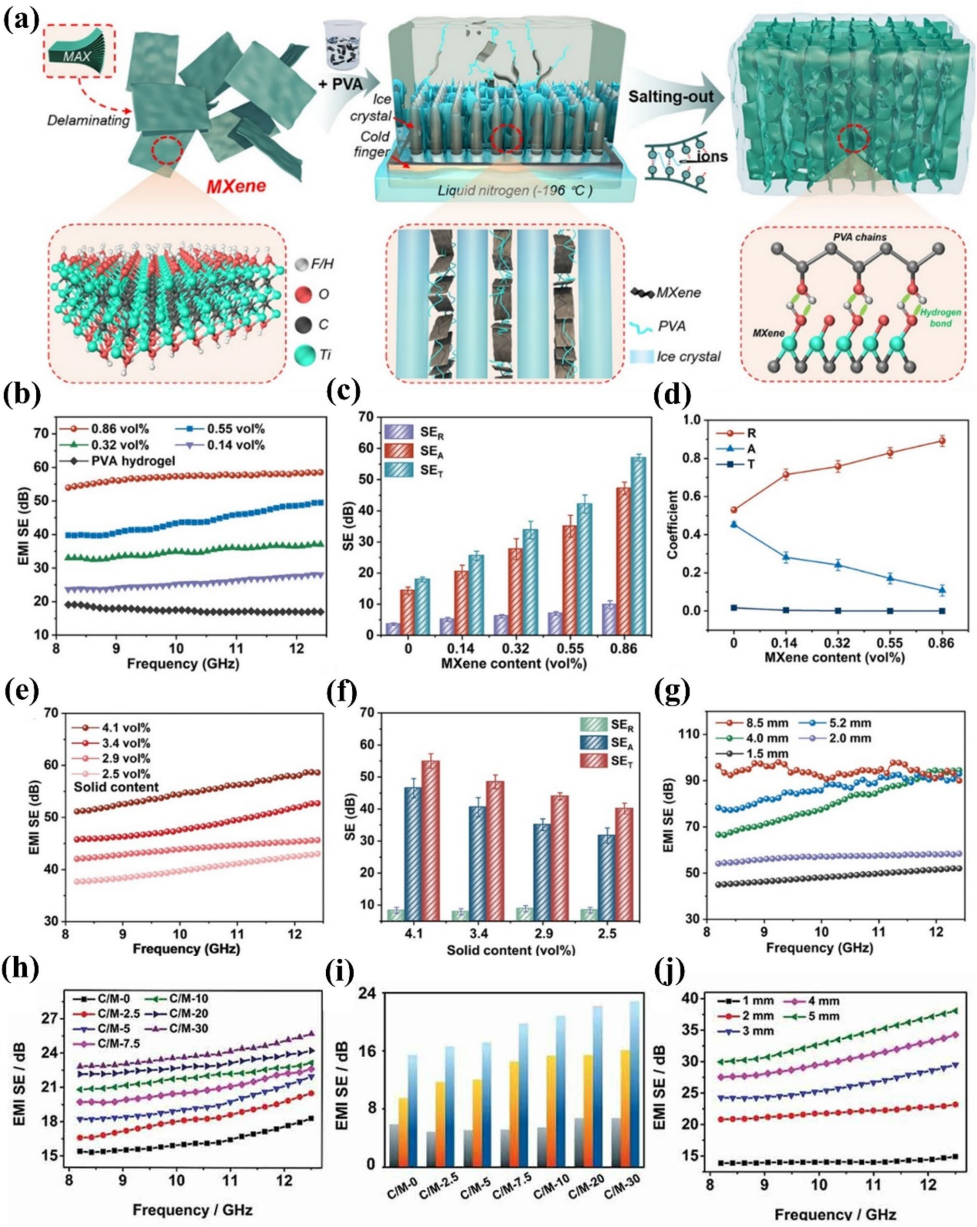
**a** Graphical description displaying the generation of MXene/PVA hydrogels. **b** X-band EMI SE and **c** SE_R_, SE_A_, and SE_T_. **d** Power parameters of hydrogels with different MXene contents. **e** EMI SE and **f** SE_*R*_, SE_*A*_, and SE_*T*_ for varying solid contents of 0.86 vol% MXene/PVA hydrogels. **g** Role of sample thickness in the EMI SEs of 0.86 vol% MXene/PVA hydrogels. **h** EMI SEs of C/M hydrogels with varying MXene loading in the frequency scope of 8.2–12.4 GHz, **i** SE_*T*_, SE_*A*_, and SE_*R*_ of hydrogels with varying MXene loading at 8.2 GHz. **j** EMI SEs of hydrogels with varying thicknesses. **a**–**g** Reproduced with consent from Ref. [60]. Copyright 2022, American Chemical Society. **h–j** Reproduced with consent from Ref. [61]. Copyright 2022, Taylor & Francis Group

Yang et al. prepared MXene/polyvinyl alcohol biomimetic hydrogels using an ice-templated freeze salting-out method (Fig. 6a) [60]. Except their great conductivity, mechanical strength, and ultraflexibility, the porous structures exhibited a honeycomb arrangement. The EMI SE of thin MXene/PVA hydrogels reached 57 dB in the X-band when the MXene content was only 0.86 vol% (Fig. 6b–g). The outstanding EMI shielding performance can be attributed to the synergistic mutual effect among MXene, PVA, water, and the biomimetic porous construction. The MXene nanosheets provided ample mobile charge carriers, which increased the conductivity of the hydrogel, leading to significant reflection losses of incident EMWs. The porous construction extended the propagation path of the EMWs, thereby ensuring extensive interactions between them and the cell walls to realize efficient EMWs depletion. Finally, the polarization relaxation of water molecules under an electromagnetic field and changes in the hydrogen bond network consumed EMWs energy. In addition, a strong dipole formed at the interface between PVA and the surface functional groups of the MXene nanosheets.

Bai et al. prepared a cellulose/MXene hydrogel that exhibited great electrical conductivity and good mechanical nature by adding MXene to a preprepared cellulose solution. First, a cellulose solution (4 wt%) was prepared by dissolving cellulose powder in an alkali (NaOH)/urea (7 wt%:12 wt%) aqueous solution, followed by freezing [61]. The great electrical electroconductibility and uniform dispersion of MXene, in the cellulose hydrogel matrix, as well as the polarization loss of water resulted in efficient EMI shielding. Meanwhile, the internal 3D porous structure provided channels for the incident wave, which further increased internal multiple-reflection attenuation and improved the shielding performance. Ultimately, the cellulose/MXene hydrogel displayed a remarkable EMI shielding performance with a EMI SE of 30 dB at 8.2 GHz (Fig. 6h–j). In addition to their excellent EMI shielding capacities, the hydrogels also exhibited impressive mechanical properties owing to the cellulose-derived 3D support framework. The rich surface functional groups of the MXene nanosheets effectively cross-link the hydroxyl groups of cellulose, delivering a cellulose/MXene hydrogel with a tensile strain of 144.4%, compressive strain of 68.7%, and Young's modulus of 9.46 kPa.

Although MXene-based hydrogel EMI shielding materials have progressed considerably, studies on the influence of pore structure regulation and the effects of individual pore components on the shielding performance are seldom reported. Thus, elucidating these mechanisms can inspire future developments in excellent hydrogel-based EMI shielding systems.

### MXene Films

#### Laminate and Composite-Structured MXene Films

MXene laminate films comprising three distinct forms have been reported [62–65]. First, the MXene nanosheets are separately produced from their MAX phases with a chemical etching technique [42]. Subsequent vacuum-assisted filtering of the aqueous MXene dispersions creates uniformly aligned MXene laminate sheets with the thicknesses of several microns.

**Fig. 7:**
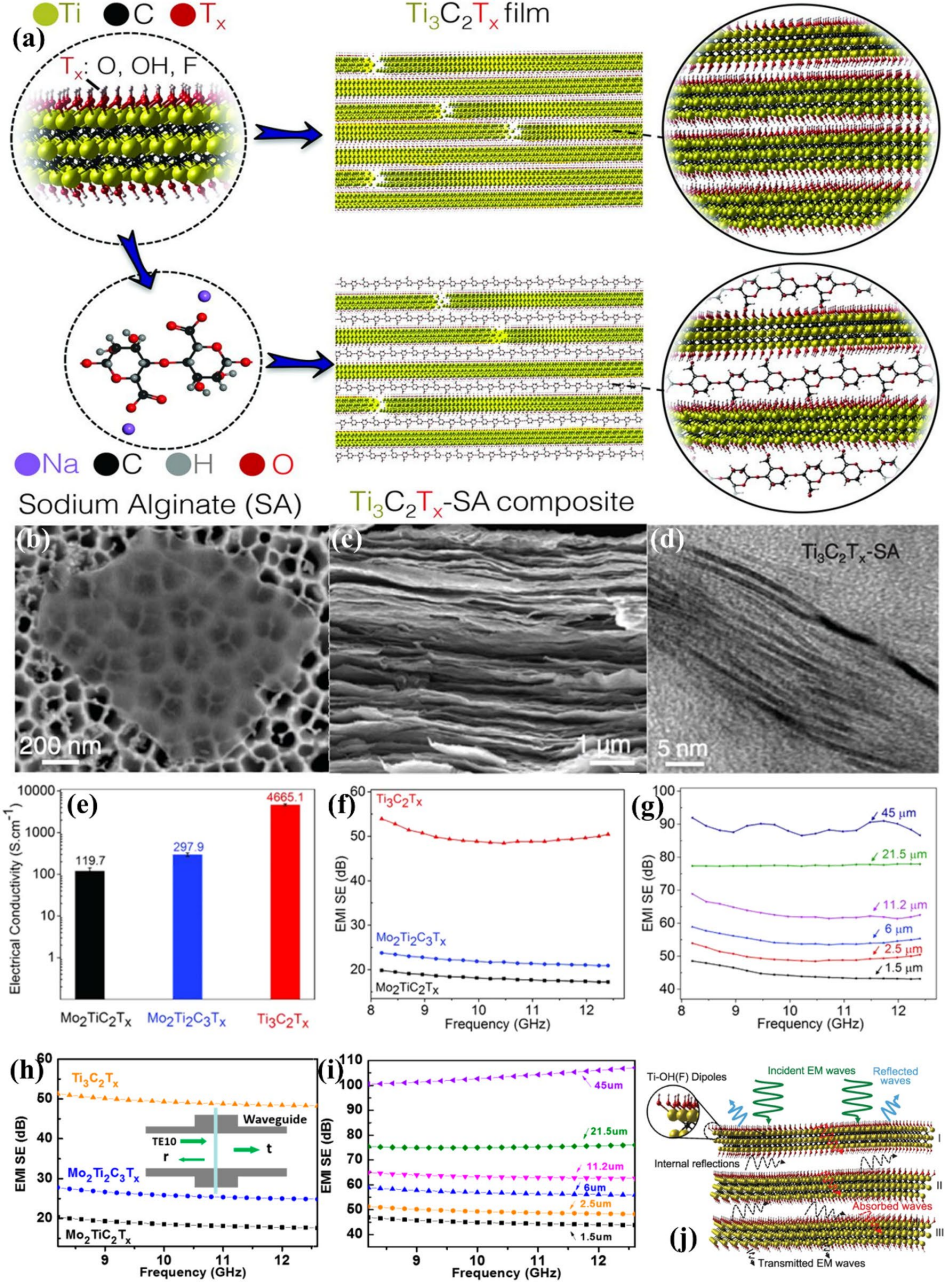
**a** Schematic of MXene and MXene-SA composite films. SEM figures of **b** MXene flakes, and **c** cross-sectional figures of a bare MXene film. **d** TEM figure of MXene-SA composite film. **e** Electrical electroconductibility and **f** EMI SE of Mo_2_TiC_2_T_*x*_, Mo_2_Ti_2_C_3_T_*x*_, and Ti_3_C_2_T_*x*_. **g** EMI SEs of MXene films of varying thicknesses. **h** Calculation of EMI SE of three MXene films; inset shows schematic drawing of waveguide measurement. **i** Calculation of EMI SEs of MXene films with varying thickness. **a–g** and **j** Reproduced with consent from Ref. [27]. Copyright 2016, Science. **h**, **i** Reproduced with consent from Ref. [28]. Copyright 2018, MDPI

The hydrophilicity of MXenes allows for the development of a biocompatible laminar MXene-SA composite film (Fig. 7a). A SEM figure of an MXene flake was displayed in Fig. 7b; the MXene flakes were almost transparent to the electron beam. The cross-sectional SEM image of the 50 wt% MXene-SA film was shown in Fig. 7c; nacre-like layered MXene stacking, resembling that of pristine MXene films, was observed for all composite ratios. Cross-sectional transmission electron microscopy figures of the MXene-SA composite films confirmed the intercalation of SA layers among the MXene flakes. In addition, at greater MXene contents, the MXene flakes were predominantly stacked, and only a few were observed to be separated from SA (Fig. 7d). MXene exhibited an extraordinary EMI shielding efficacy because of its exceptional electrical electroconductibility of ≈ 5000 S cm^−1^, which was considerably higher than those of Mo_2_TiC_2_T_*x*_ and Mo_2_Ti_2_C_3_T_*x*_ (Fig. 7e). This research proved that electrical electroconductibility is a significant element in shielding (Fig. 7f). MXene demonstrated a great EMI SE of 48–92 dB (8.2–12.4 GHz), despite an increase in the mean thickness from 1.5 to 45 μm (Fig. 7g). Overall, MXene was shown to be an exceptionally suited material for EMI shielding usage. The MXene-SA composite films exhibited great EMI SE because of the great electrical conductivity induced by MXene and the laminate architecture constructed by the standardization of 2D MXene flakes. The portion of EMWs that entered the MXene-layered structure were attenuated in the form of eddy currents, ohmic losses, etc., which is due to the extensive contact between incident waves and high-electron-density MXene layers (Fig. 7j). A previous study corroborated the excellent EMI shielding properties of MXenes by presenting theoretical calculations based on the Fresnel formula and the attenuation rule. Thus, the tremendous potential of MXene as an EMI shielding material was demonstrated by the agreement between the theoretical calculations and experimental results (Fig. 7f–i).

Except reflection and absorption losses, various EMWs reflections also make contributions to the EMI shielding effect. To elucidate the roles of various reflections in the shielding capability of nanometer-thick MXene laminate sheets, Yun et al. prepared a big-area monolayer MXene film via interfacial self-assembly [44]; meanwhile, a multilayered film was created via the successive stacking of monolayer films. The monolayer MXene film demonstrated a EMW transmittance of over 90%, and exceptional flexibility. The absorbance grew, and the sheet resistance declined as the number of MXene layers grew; however, the EMI shielding effect steadily grew. Specifically, the monolayer MXene film (2.3 nm) had an EMI SE of 1 dB. The annealed sheet had SSE/t of 3.89 × 10^6^ dB cm^2^ g^−1^ maximumly. A detailed study of the shielding process was performed applying two distinct models. Simon's formula only occupies reflection and absorption, whereas the transfer matrix technique takes into account all possible shielding processes, such as SE_*M*_, SE_*R*_, and SE_*A*_. According to Simon's formula, μm-thick MXene films demonstrated a linear growth in SE_*T*_ if the film thickness was greater than 1.5 μm. Nevertheless, when the film thickness was smaller than the skin depth (7.86 μm for MXene), Simon's theory failed to predict the acquired data, demonstrating the significant impact of repeated EMWs reflection in thinner films. The non-reflected waves undergo reflection and attenuation repeatedly inside the shielding material until they are either transmitted or completely attenuated, resulting in reduced SE_*T*_, higher SE_*A*_, and lower SE_*R*_ contributions. Vacuum filtering techniques or interfacial assembly of 2D MXene flakes can be applied to generate tailored architectures inside the MXene laminates, resulting in enhanced attenuation of incoming EMWs within the structure. Electrically conductive MXene laminate nanofilms operate via the same absorption-dominating shielding mechanism as electrically conductive films with micrometer-scale thicknesses (SE_*A*_ > SE_*R*_). The repeated EMWs reflections induced by electrically conductive films and the efficient shielding performance at nanoscale thickness offer promise for shielding undesirable EMWs in sophisticated, highly integrated, and lightweight intelligent electronic systems.

**Fig. 8:**
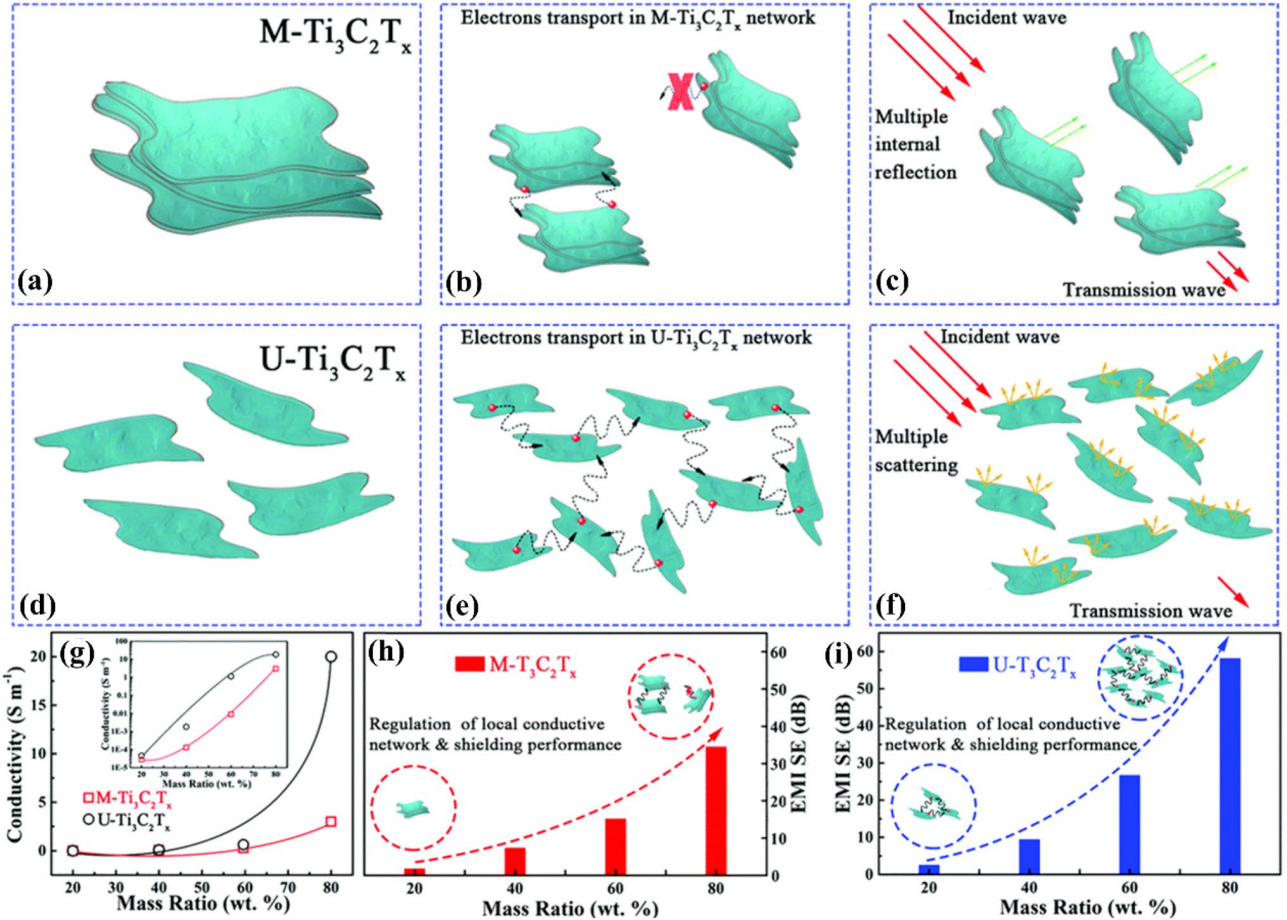
**a** M-Ti_3_C_2_T_*x*_ and **b** U-Ti_3_C_2_T_*x*_ microstructures. **c** M-Ti_3_C_2_T_*x*_ and **d** U-Ti_3_C_2_T_*x*_ local conductive networks. Microwave publicity model in **e** M-Ti_3_C_2_T_*x*_ and **f** U-Ti_3_C_2_T_*x*_ composites. **g** DC electroconductibility (*σ*) vs. mass proportion of M-Ti_3_C_2_T_*x*_/SiO_2_ and U-Ti_3_C_2_T_*x*_/SiO_2_ composites; inset displays log 10 DC electroconductibility (*σ*) vs. mass proportion. Local conductive network and shielding effect regulation of **h** M-Ti_3_C_2_T_*x*_ and **i** U-Ti_3_C_2_T_*x*_. Reproduced with consent from Ref. [66]. Copyright 2019, Royal Society of Chemistry

Depending on different etching process, MXene nanosheet layers with dissimilar thicknesses can be obtained, which may also influence the EMI shielding performance. Figure 8 shows a comparison of two distinct preparation methods [66]. Both protocols took part in the etching of Ti_3_AlC_2_ MAX powder, the first one adopted 40% hydrofluoric acid to produce an accordion-like multilayered (M-MXene) morphology, and the second one adopted LiF and HCl for 16 h at 40 °C to produce delaminated ultrathin MXene sheets (U-MXene). The M-MXene composite contained more F terminations, whereas the U-MXene composite had more = O terminations. Both MXenes were cold pressed at 5 MPa after being combined with SiO_2_ nanoparticles as an EMWs-transparent matrix at various mass proportions (20, 40, 60, and 80 wt%). As shown in Fig. 8g, the corresponding conductivities (*σ*) of the 60 wt% M-Ti_3_C_2_T_*x*_ and U-Ti_3_C_2_T_*x*_ composites were 6.3 × 10^−3^ S m^−1^ and 0.42 S m^−1^, respectively. In the 8.2–12.4 GHz frequency scope, the 1 mm-thick 80 wt% U-MXene composite attained an EMI SE of 58 dB (Fig. 8h, i). With their better electrical conductivity, bigger surface area, and more extensive conductive network, the U-MXene composites outperformed M-MXene in terms of EMI shielding. Additionally, the exposed surface areas of the U-MXene composites exhibited copious surface terminations and point deficiencies.

Similarly, Li et al. showed the affect of the etching process on enhencing the EMI shielding effect. They obtained lamellar Ti_2_CT_*x*_ MXene using a slightly modified LiF/HCl etching technique to process a Ti_2_AlC MAX phase [20]. Ti_2_AlC and laminar Ti_2_CT_*x*_ MXene were composited in a paraffin wax matrix, with the mass ratio of MXene. The electrical conductivities and EMI shielding nature of the obtained composites were examined. Although the electrical electroconductibility of Ti_2_CT_*x*_ MXene is lower in comparison with that of pure Ti_2_AlC MAX, the electrical electroconductibility values of the Ti_2_CT_*x*_ MXene-based composites were greater than those of the composites based on Ti_2_AlC MAX at 40 wt%. The electrical electroconductibility of the Ti_2_CT_*x*_/paraffin composite and Ti_2_AlC/paraffin composites were 1.63 × 10^–8^ and 5.57 × 10^–9^ S cm^−1^. The exfoliated Ti_2_CT_*x*_ MXene sheets had a large surface region, allowing the effective formation of a robust conductive network even at low mass proportions. Therefore, the great electrical electroconductibility of delaminated Ti_2_CT_*x*_ MXene resulted in a greater EMI SE (70 dB) by comparing with that of the Ti_2_AlC MAX phase at thickness > 0.8 mm.

As mentioned previously, the high electrical conductivity of MXene laminates accounts for their outstanding EMI SE. Nevertheless, in real applications, the smaller size of the 2D MXene sheets may result in unfavorable mechanical characteristics and poor oxidation resistance. Consequently, the production of great and resistant MXene hybrids and composites has increased significantly. The homogeneous dispersion of nanowires, 1D nanofibers, or polymeric chains into 2D MXene nanosheets enhances the mechanical strength and EMI SE of composite films. Under strain, nanofibers and nanowires may operate, while polymers induce the formation of brick-and-mortar structures to provide flexible, robust, and mechanically strong structures. Moreover, the addition of polymeric chains can also enhance the oxidation resistance of composites, making MXene-polymer composites a promising choice. Cao et al. fabricated MXene-cellulose nanofiber composites with nacre-like structures and tested them using a simple vacuum-assisted filtering process [67]. The dispersion of the 1D nanofibers led to fewer insulated connections between the 2D MXene nanosheets. At 12.4 GHz, a nanocomposite containing 80 wt% MXene displayed an electrical electroconductibility of 1.155 S cm^−1^ and an EMI SE of 25.8 dB. The robust nanofibers enhanced the mechanical nature of the composite films, which were superior to those of pure MXene and CNF paper. At a MXene content of 50%, the ultimate tensile strength was 135.4 MPa and fracture appeared at 16.7% strain. The mother-of-pearl construction of the composite film originated from great hydrogen bonding among the hydroxyl groups on the CNF and MXene surfaces, which also contributed to its excellent strength. Hydrogen bonding enables the composite to withstand higher stresses as it maintains a well-stacked and compact morphology. Therefore, MXene composites are promising candidates for use in foldable and wearable electronics. Xie et al. capitalized on the favorable mechanical properties of polymers to develop an ultrathin MXene-aramid nanofiber (ANF) composite for EMI shielding [68]. The final tensile strength and fracture strain of the ANF paper increased to 197 MPa and 9.8%, at low MXene concentrations of < 10 wt%. The electrical electroconductibility and EMI SE MXene composite film were 170 S cm^−1^ and 33 dB. He et al. researched the EMI shielding efficiency of Ti_3_C_2_T_*x*_/hydroxyethyl cellulose nanocomposites [69], in which the confined organic HEC chains increased the mechanical strength of the composite by generating hydrogen bonds with the hydroxyl termini of MXene. In the observed X-band frequency scope, MXene/HEC composite of 100 μm thickness revealed an EMI SE of 24 dB. Wang et al. adopted a solution casting process to build low-temperature annealed MXene/epoxy composites [70]. At 2 mm thickness, the electrical electroconductibility of the insulating epoxy was increased to 0.38 S cm^−1^ by unannealed MXene, and 1.05 S cm^−1^ by MXene, which resulted in EMI SEs of 30 and 41 dB, respectively.

The introduction of insulating polymers may affect the total electrical conductivity of the material and diminish the EMI shielding ability; therefore, conductive polymers have attracted increasing attention. The use of conductive polymers preserves electrical conductivity. Conductive chains among MXene layers provides a conductive route for electron transport. Liu et al. prepared vacuum-screened MXene and PEDOT [71]. The mother-of-pearl-type composite exhibited high electrical conductivity owing to its brick-and-mortar construction, where 2D MXene sheets could be taken as bricks and the conductive organic polymer chains as mortar. The conductivity of the composite with an MXene/PEDOT: PSS proportion of 7:1 (340.5 S cm^−1^) was lower than that of pure MXene, while the EMI SE values for both materials were 42 dB, and the composite was 1.94 g cm^−3^ dense. The composite construction preserved the electrical properties of MXene, allowing for the attenuation of EMWs by different polarization processes, which showed relatively poor conductivity. Similarly, Zhang et al. examined the EMI shielding nature of ultrathin MXene/polyaniline (MXene/PANI) composites [72]. Sufficient electrical electroconductibility was realized because of the conductive polymeric chains among the MXene layers. Consequently, an EMI SE of 36 dB was obtained at 40 μm thickness.

Wang et al. prepared core–shell Fe_3_O_4_@PANI composites, and then flexible lightweight Ti_3_C_2_T_*x*_/Fe_3_O_4_@PANI composite membranes with intercalated intercalation construction by vacuum-helped filtration applying Ti_3_C_2_T_*x*_ MXene as the matrix material [131]. When the Ti_3_C_2_T_*x*_ nanosheets have a 12:5 weight-to-Fe_3_O_4_@PANI ratio, EMI shielding efficiency of 58.8 dB maximumly can be achieved with a thickness of only 12.1 μm, which is greater than the SE of MXene/PANI films of the same thickness. After modifying the film thickness by growing the concentration of Ti_3_C_2_T_*x*_, a SE of 62 dB can be realized at a thickness of 16.7 μm. Magnetic Fe_3_O_4_ particles bring about the favorable magnetic loss, thus the electromagnetic wave absorption effect of the composite is enhanced through natural resonance and exchange resonance. Meanwhile, the multiple reflection and attenuation of internal electromagnetic waves are also increased through structural design. Therefore, the addition of semiconductor metal oxides and the design of the structure exert an important influence on improving the electromagnetic shielding effect of the material.

**Fig. 9:**
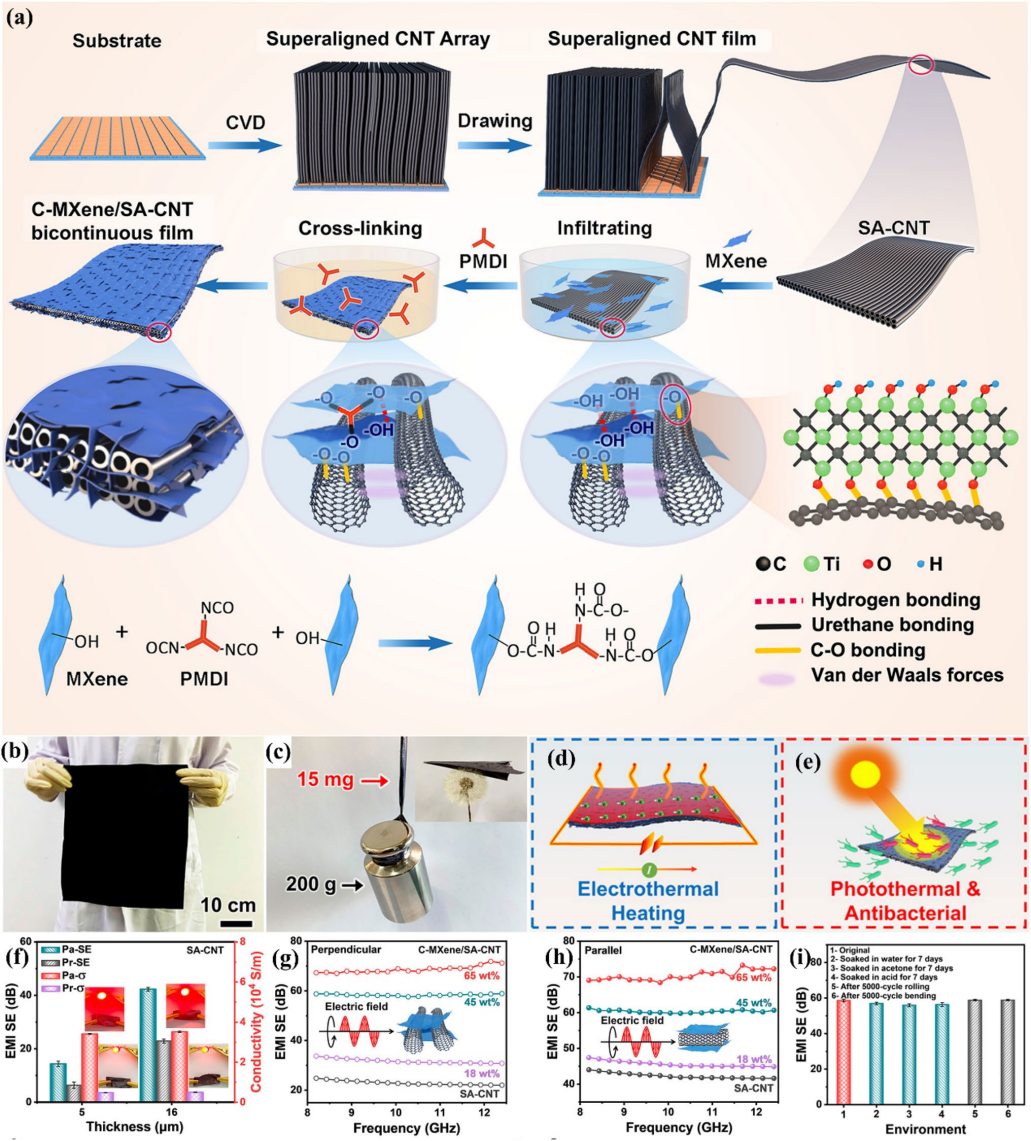
**a** Schematic showing the generation of C-MXene/SA-CNT films. **b** Figure of big freestanding C-MXene/SA-CNT films. **c** Figure of a 200 g weight suspended from a 15-mg C-MXene/SA-CNT film (inset displays an origami plane). **d** Electrothermal and **e** photothermal nature of C-MXene/SA-CNT films. EMI shielding effect of the C-MXene/SA-CNT films. **f** EMI SE and electrical electroconductibility of SA-CNT films of different thicknesses in perpendicularly and parallelly. EMI SE in the X-band of C-MXene/SA-CNT films with different MXene contents **g** perpendicularly and **h** parallelly. **i** X-band EMI SEs of the films after 7-day soaking in different solvents or after 5000-cycle bending at a bending speed of 0.5 Hz and rolling. Reproduced with consent from Ref. [93]. Copyright 2020, American Chemical Society

MXene hybrid films can be formed to improve the EMI shielding efficiency [91, 92]. Li et al. manufactured lightweight, thin, large-area, and ultra-flexible bicontinuous chemically cross-linked MXene/superaligned carbon nanotube composite sheets (Fig. 9a–c) [93]. The films exhibited great mechanical strength, strong electrical electroconductibility (Fig. 9f), hydrophobicity, oxidation steadiness (Fig. 9i), wearable multifunctionality, EMI shielding (Fig. 9f–h), electrothermal conversion (Fig. 9d), and photothermal antibacterial properties (Fig. 9e). The X-band EMI SE ranged from 24 to 70 dB as the thickness increased from 8 to 28 μm in the ultrabroadband frequency scope of 8.2–40 GHz. An SSE/t of 122,368 dB cm^2^ g^−1^ was obtained, greatly exceeding those of other reported shields. The excellent electro/photothermal effect of the films resulted in effective de-icing and antibacterial characteristics. Integrated with an economical and scalable production process, the multifunctional wearable bicontinuous films display enormous promise for usage in wearable equipment, defense systems, antibacterial devices, and the Internet of Things.

Cao et al. created a CNT/MXene/CNF composite film [94]. They examined the synergistic role of conductive CNTs and CNFs in the EMI shielding and mechanical characteristics of the composite films. The resulting composite film exhibited remarkable mechanical characteristics. Because of the synergistic role of conductive CNT-MXene and nonconductive CNFs, the CNT/MXene/CNF hybrid film displayed a great electrical electroconductibility.

Xin et al. developed a MXene/CNF/Ag composite material for EMI shielding usage [95]. A vacuum filtration process was used to create the MXene/CNF/Ag composite film, in which MXene yielded silver nanoparticles. The composite material displayed a high electrical electroconductibility of 588.2 S m^−1^ and an excellent EMI SE of approximately 50.7 dB, which was considerably greater than that of a MXene/CNF layer of similar thickness.

Wang et al. developed a PVDF/MXene/Ni composite film and studied the synergistic impact of MXene and Ni chains on the EMI SE [96]. A PVDF/MXene/Ni composite film of 0.3 mm thick displayed an electrical electroconductibility of 892 S m^−1^ and an EMI SE of 34.4 dB, both of which were higher than those of pure MXene or pure Ni chains with the same loading. The mechanical properties were also examined. The Young's modulus, tensile strength, and toughness of the PVDF/MXene/Ni chain composite films were approximately 1.18 GPa, 41.9 MPa, and 2.9 MJ m^−3^.

Xiang et al. [97] generated a TiO_2_-MXene/graphene film for EMI shielding via vacuum screening and pyrolysis. First, the fabrication of MXene/graphene film was made via vacuum filtration, followed by heat treatment for the partial transformation of MXene to TiO_2_ to generate a TiO_2_-MXene/graphene film. The composite material showed a low surface resistance of 7.5 V (sq)^−1^ and an outstanding EMI SE of > 27 dB. Furthermore, while taking into account the density and thickness of the shielding film, the TiO_2_-MXene/graphene film exhibited an astounding shielding effect^1^.

Liu et al. manufactured GO-enhanced MXene films with outstanding electrical conductivities and mechanical strength using vacuum-helped filtering [98]. The acquired MXene/GO film exhibited an exceptional tensile strength of 209 MPa, which is approximately nine-fold greater than that of the pure MXene film. In the meantime, the composite film exhibited an electrical electroconductibility of 2.64 × 10^5^ S m^−1^, which was comparable to that of the MXene film. The superior electrical electroconductibility of the MXene/GO film resulted in an exceptional EMI SE of 50.5 dB at a film thickness of 7 mm. Besides, the comparison of MXene-GO films were made with analogous shielding films described in the literatures with respect to SE vs. strength.

Miao et al. fabricated an AgNW/nanocellulose/MXene composite film using a pressure extrusion film-forming method [99]. In this technique, NC was first disseminated across 2D MXene nanosheets through hydrogen bonding. Subsequently, the intercalation of the AgNWs formed a greatly interpenetrating 1D/2D comprehensive conductive network. The composite film demonstrated a phenomenal electroconductibility of 30,000 S m^−1^ and an exceptional EMI SE of 16,724 dB. The tensile stress of the AgNW/NC/MXene composite film was 63.80 MPa, which was approximately 49.5-fold higher than that of the pure MXene film. Besides, after 10,000 bending cycles at 135 °C, the electroconductibility and EMI SE of the composite film remained stable.

#### Layer-by-Layer (LBL)-Structured MXene Film

The term "LBL-constructed film" means a film comprising alternating nanometer-thick monolayers of oppositely varied substances assembled via successive absorption. LBL films constructed using two phases with differing impedances may trigger significant internal scattering at the internal interfaces. Spin coating [73], spray coating [74], dip coating [75], solution casting [76], and interfacial assembly are common solution processing methods used to build LBL structures [77]. The hydrophilic surfaces of exfoliated MXene sheets allow for the preparation of steady dispersions in aqueous and organic solvents, allowing solution treatment of a variety of nanostructures and polymers [78]. Mechanical strength can also be increased by employing an LBL structure.

**Fig. 10:**
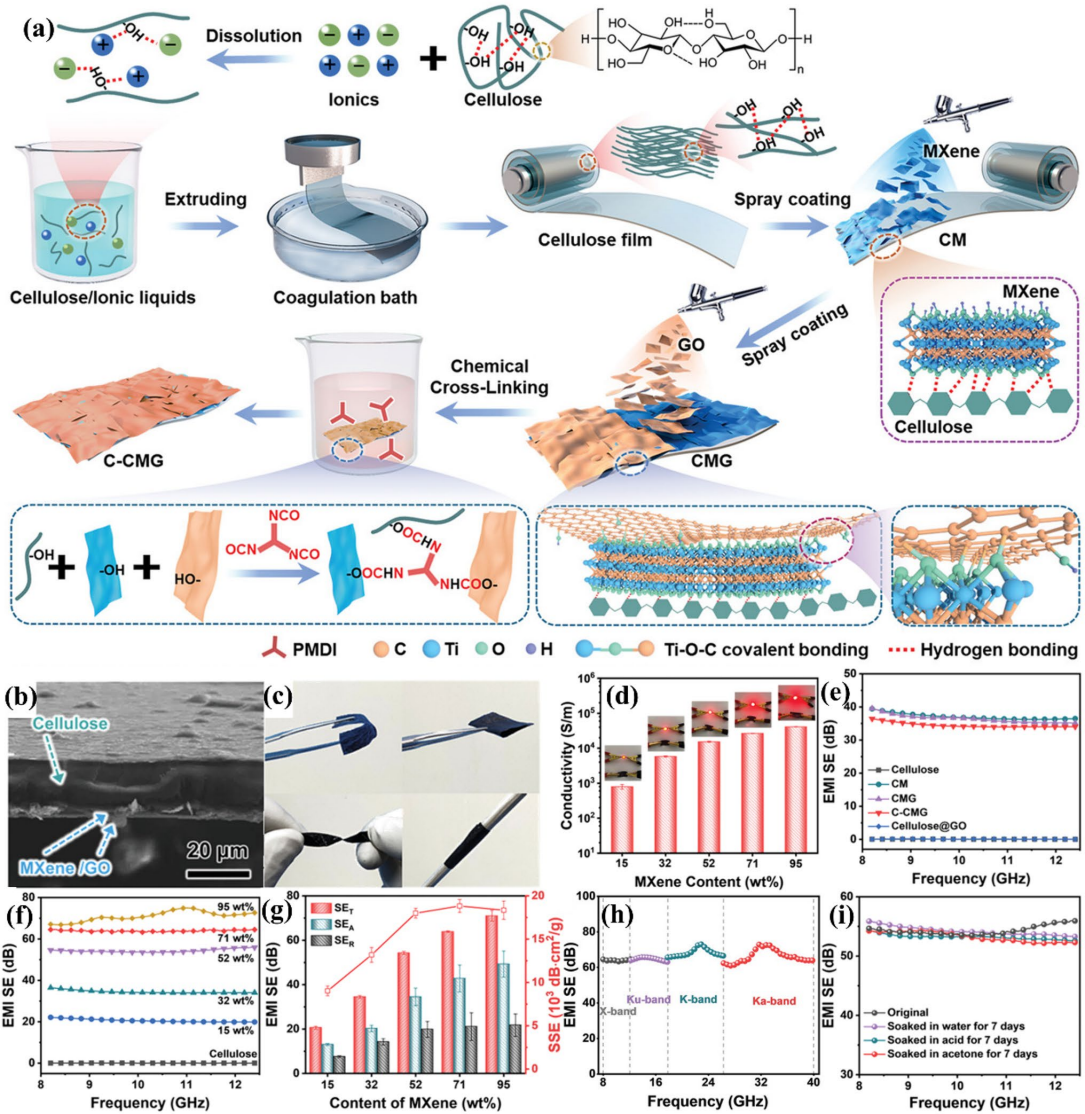
**a** Schematically showing the producing process of C-CMG films. **b** Cross-sectional SEM figure of the C-CMG film. **c** Figure of a C-CMG film showing ultra-flexibility. **d** Electrical conductivities of C-CMG films with varying MXene contents. EMI shielding performances of the C-CMG films. EMI SEs in the X-band of **e** cellulose, CM, CMG, C-CMG, and cellulose@GO films and **f** C-CMG films with MXene contents. **g** EMI shielding performances (SE_T_, SE_A_, and SE_R_) at 10 GHz and SSEs of the C-CMG films with varying MXene contents. **h** EMI SE values of the 30 μm-thick C-CMG films. **i** X-band EMI SE of the films after 7-day soaking in various solvents. Reproduced with consent from Ref. [79]. Copyright 2022, Wiley–VCH

For instance, Li et al. produced big, high-strength, ultra-flexible hybrid films using graphene oxide to promote the physical and chemical cross-linking of MXene sheets and cellulose (Fig. 10a) [79]. The hydrophobicity, water/solvent resistance, and oxidative steadiness of MXene-based films were significantly enhanced (Fig. 10c, h). Favorable conductivities (Fig. 10d) and EMI shielding performances were achieved (Fig. 10e–g). An X-band SSE/t of 18,837.5 dB cm^2^ g^−1^ and an EMI SE exceeding 60 dB were attained, which is equivalent to the best performances reported to date (Fig. 10h). Additionally, the photothermal, antibacterial, and electrothermal de-icing capabilities of the composite films were shown. Consequently, such effective MXene-based films prepared by simple and scalable manufacturing approaches offer significant application possibilities.

In addition to graphene, highly conductive carbon nanotubes (NTs) can also be applied for constructing LBL-structured composite films with excellent EMI shielding capabilities. Weng et al. developed a cross-functional semi-transparent Ti_3_C_2_T_*x*_-carbon nanotube (MXene/CNT) composite film with an LBL structure using the spin-spray approach and investigated its EMI shielding ability [80]. The developed MXene/CNT composite film showed a high degree of transparency and is applicable in various industries. The electrical electroconductibility of the 300-bilayer composite film was 130 S cm^−1^ and the SSE/t was 58,187 dB cm^2^ g^−1^. These were among the greatest values observed in flexible and semi-transparent composite thin films. Surprisingly, EMWs absorption was the primary shielding system of the as-prepared MXene/CNT composite films. The excellent shielding properties were largely derived from the LBL structure of the film.

The mismatched impedance of the multilayered stacked LBL architecture with alternating conducting and nonconducting layers improved the internal scattering and absorption of EMWs. Jin et al. created a PVA/MXene composite film using a simple liquid casting process and LBL assembly of Ti_3_C_2_T_*x*_ MXene and polyvinyl alcohol (PVA) [81]. The initial layer is constructed by the application of an aqueous PVA solution to a clean iron substrate, next to drying at 45 °C. Under the same conditions, MXene layers of differing concentrations were deposited on the PVA layer and cured. This procedure was repeated until the desired thickness was achieved, after which, a final PVA coating was deposited. Varying the number of stacking cycles yielded various Ti_3_C_2_T_*x*_ MXene contents (7.5, 13.9, and 19.5 wt%). Owing to the combination of PVA and Ti_3_C_2_T_*x*_ MXene, the multilayered compacted films with single-layer thicknesses of 1–3 μm demonstrated outstanding mechanical flexibility. The electrical electroconductibility of the composite films increased with increasing Ti_3_C_2_T_*x*_ MXene concentration, reaching 7.16 S cm^−1^. The EMI SE followed a similar trend, attaining 44.4 dB maximumly at the highest Ti_3_C_2_T_*x*_ MXene content and a thickness of 25 μm.

CNFs are also suitable for LBL assembly because their functional groups can bond to MXene to form a stable connection. Zhou et al. adopted a simple and effective alternating vacuum filtration approach to manufacture MXene/CNF composite films with an LBL construction [82]. Because of the mechanical frame role exerted by the CNF layers, this alternating multilayered film outperformed both freestanding MXene and homogeneous CNF/MXene films in terms of mechanical strength and toughness. Moreover, the LBL MXene/CNF films displayed great electrical conductivities of 82–621 S m^−1^. Surprisingly, the in-plane electrical electroconductibility was almost nine orders of magnitude greater than that in the through-plane orientation. This anisotropic electrical electroconductibility implies promising potential for insulation-requiring EMI shielding applications. Most impressively, the LBL MXene/CNF film showed a remarkable EMI SE, as well as a high SSE/t of 7029 dB cm^2^ g^−1^ at just 35 μm thickness. The LBL construction comprising conductive and non-conductive layers induced extensive "reflection–absorption zigzag" reflection, thereby considerably enhancing the EMI SE.

LBL structures with tailored chemical, mechanical, and electrical properties can be applied in a broad range of fields. Moreover, the shape of the conducting material is critical for lowering the skin effect [80, 83] and enhancing EMWs interactions within the layers via internal scattering, thereby significantly affecting EMI shielding properties. The advantages of MXene-based LBL composites include their adaptable density, and favorable electrical characteristics as well as mechanical strength, allowing large-scale regulation of EMI SE. allowing for EMI SE.

#### Fabric-Structured MXene Films

Flexible fabrics with conductive and EMI-shielding properties have drawn increasing interest in wearable and portable electronics. Except providing electrical electroconductibility, thermal electroconductibility, and EMI shielding nature, the integration of conductive fillers and fabric retains the stretchability, flexibility, and breathability of the fabric. MXenes, which are highly conductive and contain abundant functional groups, are ideal conductive fillers for electrical fabrics [84–87].

**Fig. 11:**
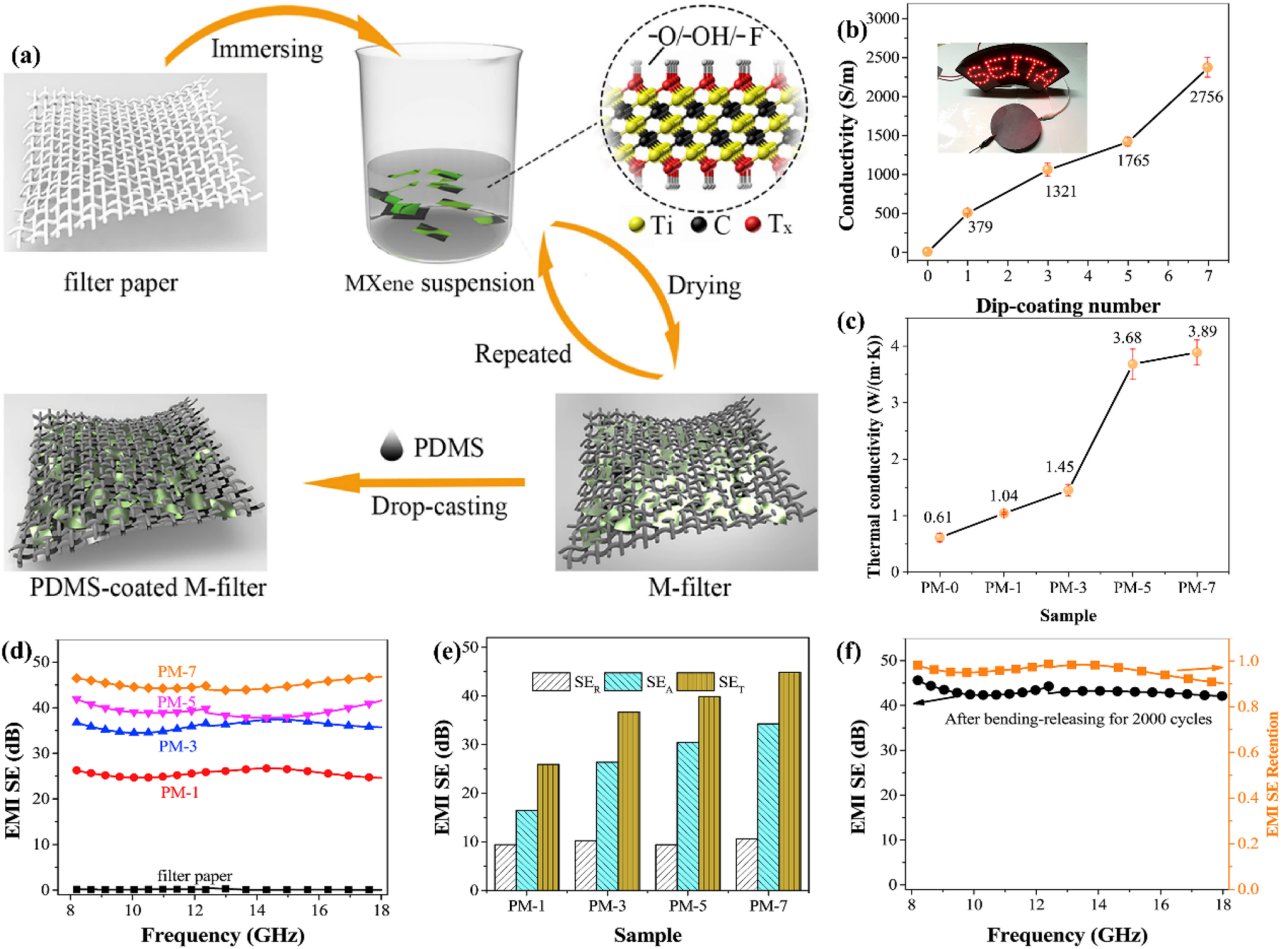
**a** Schematic description of the production process of freestanding nanocomposites. **b** Electrical electroconductibility of M-filter. **c** Thermal electroconductibility of M-filter. **d** EMI SE of the filter paper and PDMS-coated M-filter. **e** Contrast of SE_T_, SE_R,_ and SE_A_ of PDMS-coated M-filters with varying dip-coating cycles at 12.4 GHz. **f** EMI SE of PM-7 after 2000 bending-releasing cycles and EMI SE retention. Reproduced with consent from Ref. [88]. Copyright 2020, Elsevier

Using commercial cellulose filter paper as the substrate, Hu et al. employed a simple dip-coating approach to generate greatly lectrically and thermally conductive, flexible, and robust MXene/cellulose films (Fig. 11a) [88]. The linked MXenes provide strong electrical and thermal electroconductibility to the composite sheets. Because of the inherent metallic conductivity of Ti_3_C_2_T_*x*_ nanosheets and their linked network, the resulting composite film showed an exceptional electrical electroconductibility of 2756 S m^−1^ (Fig. 11b). Furthermore, the EMI SEs of the MXene/cellulose films reached 43 dB in the X- and Ku-bands (Fig. 11d, e). Notably, the composite material was mechanically stable. Moreover, the composite sheet showed an in-plane thermal electroconductibility of 3.89 W m^−1^ K^−1^ (Fig. 11c).

Several kinds of textile materials have been manufactured using solvent-based processes including dip, spray, vacuum filtering, and screen printing. Two-dimensional MXenes exhibit good physicochemical characteristics and are regarded as appropriate nanomaterials for the fabrication of multi-functional flexible textiles. The findings showed that the synthesized MXene composite textiles have good shielding characteristics. The textiles also showcase multiple functionalities, such as hydrophobicity, heat dissipation, and so on, significantly expanding the application range of MXene fabrics.

Liu et al. employed vacuum-assisted LBL spray coating to develop MXene and silver nanowire -doped silk textiles, which displayed excellent EMI shielding, superhydrophilicity, and a greatly sensitive humidity reaction [89]. The leaf-like construction of the composite film consisted of MXene and AgNWs. This multifunctional film had a low sheet resistance of 0.8 V (sq)^−1^, an excellent EMI SE of 54 dB in the X-band, and a thickness of 120 μm, while maintaining acceptable porosity and penetrability. Similarly, Zhang et al. applied a simple spray-drying coating process to create MXene-decorated woven cotton textiles [87]. By modifying the spray-drying cycle, the degree of conductive connections in the fabric could be accurately changed; thus, the conductivity and EMI SE were readily controllable. Owing to its interwoven conductive architecture, M-CF exhibited a decent resistance of 5 V (sq)^−1^ at a modest loading of 6 wt%. Furthermore, the obtained MXene-decorated textiles exhibited satisfactory EMI SEs of above 36 dB at a low MXene loading (6 wt%). In addition, M-CF exhibited an excellent Joule heating capacity and kept the natural air penetrability and elasticity of the fabric.

Ma et al. used a simple "dipping and drying" process to yield a MXene-decorated air-laid paper with a superb folding endurance and EMI shielding effect [90]. In the "dipping and drying" process, the air-laid papers are coated by MXene as a result of van der Waals forces and hydrogen bonding between the MXene nanosheets and cellulose. The MXene-decorated paper showed a great electrical electroconductibility of 173.0 S m^−1^, excellent EMI shielding efficiency, substantial mechanical flexibility (it could be folded 9.8 × 10^4^ times), and breathability.

### MXene-Based Materials with a Segregated Structure

For conductive polymer composites with a unified construction, a high filler content is often required because inefficient lapping of the conductive filler inhibits the formation of a conductive network. To construct a more complete conductive network with a low proportion of conductive filler, an internal conductive network has been designed as a segregated construction. The preparation of conductive composites with a segregated structure generally entails the attachment of conductive fillers to the surface of polymer particles (similar to a core–shell structure), and then combining the materials under pressure to form a material with a segregated structure. The selective distribution of conductive fillers is made between the microinterfaces of the polymers, increasing the probability of lapping between the conductive fillers.

**Fig. 12:**
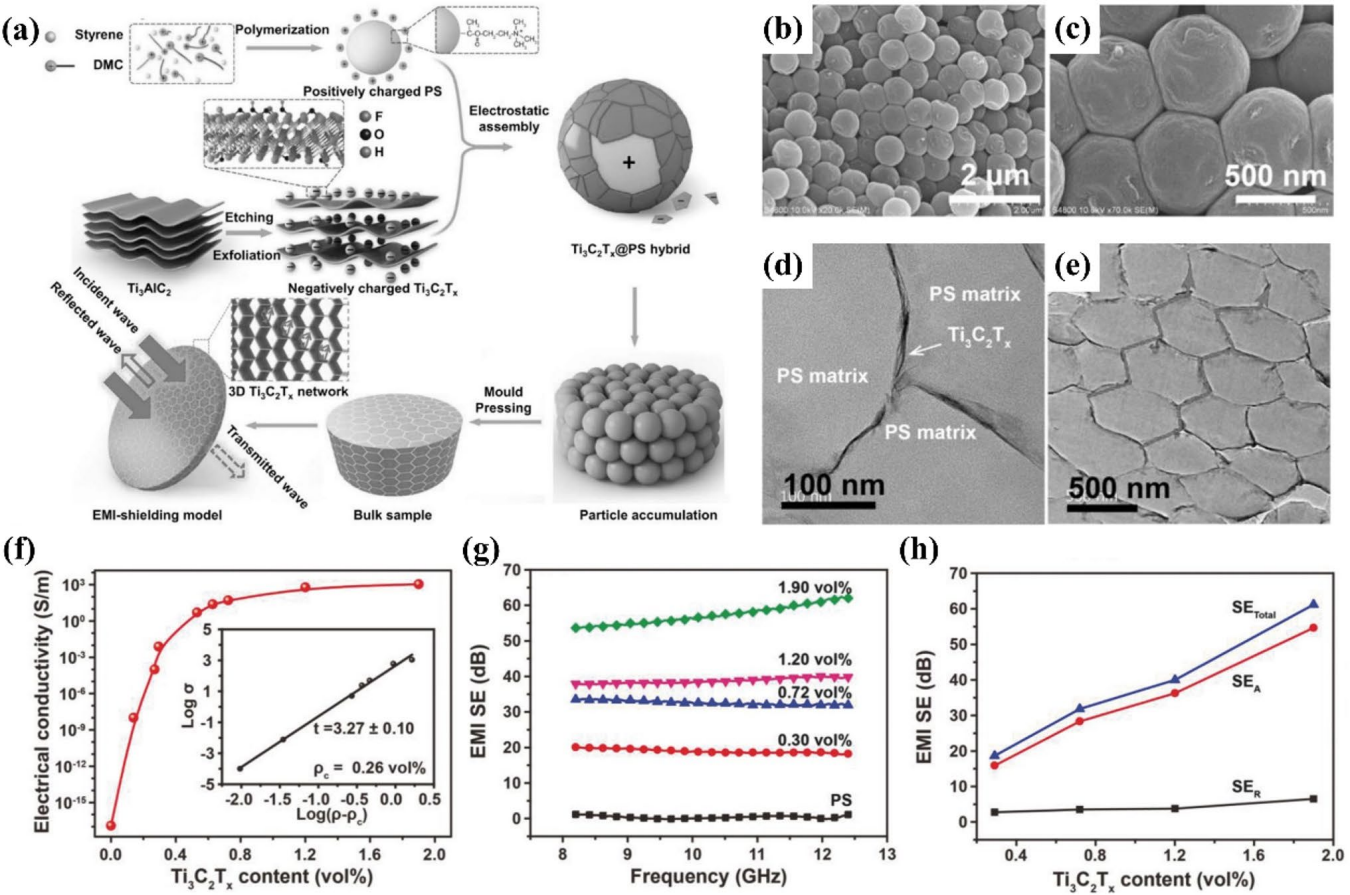
**a** Schematically describing the generation of Ti_3_C_2_T_*x*_@PS nanocomposites. **b**, **c** SEM figures of cross section of Ti_3_C_2_T_*x*_@PS-570 hybrid made at ambient temperature. **d**, **e** TEM figures of Ti_3_C_2_T_*x*_@PS-570 nanocomposites with 1.90 vol% of Ti_3_C_2_T_*x*_; dark gray lines and white arrows mean electrical conduction paths. **f** Electrical conductivity, **g** EMI SE and **h** SE_T_, SE_A_, and SE_R_ of Ti_3_C_2_T_*x*_@PS-570 nanocomposites. Reproduced with consent from Ref. [100]. Copyright 2017, WILEY–VCH

Zhang et al. made a greatly conductive MXene@polystyrene (PS) nanocomposite material (Fig. 12) [100]. Due to the great electroconductibility of MXene and the continuous and effective conductive network formed inside the PS matrix, the obtained nanomaterials showed a low permeability threshold of 0.26 vol% and great electroconductibility of 1081 S m^−1^, and achieved an excellent EMI SE of > 54 dB over the entire X-band. At a low MXene loading of 1.90 vol%, the EMI shielding effect of a 2 mm-thick nanocomposite was 62 dB. The elasticity modulus of the material was 54% and 56% greater than those of pure PS and traditional MXene@PAA nanocomposites. By adjusting the PS diameter, the conductivity of the MXene@PS nanocomposite material could be controlled; under the same load, MXene nanosheets favored the assembly on the surface of large microspheres to form a more continuous network, which was also conducive to load transfer and improved conductivity and the elastic modulus. Eventually, the continuous 3D conductive network formed at the interface of the hexagonal PS substrate, facilitated the dissipation of EMWs.

Such a structural design can also reduce the density of the system and impart other favorable properties, such as heat insulation, sound absorption, and high elasticity, by prefabricating the matrix, using methods such as supercritical CO_2_ foaming, or by introducing a cell structure after the isolation structure is completed. Therefore, shielding materials with segregated structures are widely used. However, the conductive filler content in the isolation structure should generally not exceed 10 wt%, otherwise the dispersion of the filler at the interface becomes difficult.

## Overview and Perspectives

The emergence of MXenes in EMI shielding in 2016 has had a transformative effect on this field. Since then, a slew of reports on "2D MXene and EMI shields" have been published. Several structural forms have been examined in terms of various performance indicators including thickness, density, and EMI SE. This review presented notable research achievements in the development of EMI shielding macrostructures based on MXene and emphasized the relevant structure–property relationships. Table 1 sums up the EMI SEs of MXene-based shields with various structures and the corresponding composition, thickness, density, and SSE/t. The data indicate that the structures of the materials have a profound effect on various performance indicators. (1) Porous foams and aerogels provide robust and ultra- lightweight characters as well satisfactory EMI shielding without sacrificing thickness, which is favorable for aerospace and military applications [2, 9, 105, 121, 129]. To reaching efficient EMI shielding, a compromise between the density and thickness is unavoidable, although an appropriate combination of both may yield an outstanding SSE/*t*. (2) Compact MXene laminates and related composites are suitable for ultrathin shielding materials that require superior mechanical qualities, such as in miniaturized electronics [9, 103, 114, 125–128]. MXene laminates deliver excellent EMI SEs at minimal thicknesses and can be fabricated at the nanoscale to fulfill commercial product needs. (3) Segregated constructions with exceptionally low MXene loadings are economical.

**Table 1:** EMI shielding performances of different structural forms on basis of MXene

Structure	Type	Materials	Thickness (mm)	EMI SE dB)	Frequency (GHz)	Density (g cm^−3^)	SSE/*t* (dB cm^2^ g^−1^)	References
Porous structure	Foam	MXene	0.06	70	8.2–12.4	0.22	53,030	[56]
		MXene/SA/PDMS	2	53.9	8.2–12.4	0.405	665	[57]
		MXene/PVA	5	28	8.2–12.4		5136	[101]
		MXene/RGO	3	50.7	8.2–12.4		36,737	[91]
		MXene/PCF	0.3	74	8.2–12.4	0.35	7200	[102]
		C-MXene@PI	1.5	60.04	8.2–12.4	0.043	9315	[103]
		MXene/PDMS	5	45.2	8.2–12.4			[104]
	Aerogel	MXene/ANF	1.9	56.8	8.2–12.4		3645.7	[105]
		MXene	2	75	8.2–12.4	0.0207	9904	[58]
		MXene/CNF	2	81.35	8.2–12.4	0.045	9039	[106]
		MXene/PVA/Ca^2+^	0.679	64.2	8.2–12.4	0.107	8640.6	[107]
		MXene/PVA	0.349	48.4	8.2–12.4	0.107	13,009.6	[107]
		MXene/PEDOT: PSS	5	59	8.2–12.4	0.01	10,841	[108]
		MXene/ANF	2.5	65.5	8.2–12.4	0.02	11,391	[109]
		MXene/RGO/Epoxy	2	56.4	8.2–12.4	0.3	940	[59]
		MXene/CNT	3	104	8.2–12.4	0.042	8253	[110]
		MXene/AgNW	2	52.6	8.2–12.4	0.049	5313	[111]
		MXene/RGO	7	83.3	8.2–12.4	0.029	4456	[58]
		MXene/CS	3	61.4	8.2–12.4	0.012	5155.46	[112]
		S@BC/MXene	2	50.8	8.2–12.4	0.062	66,943.8	[113]
		Co/C/MXene/CNF	2	86.7	8.2–12.4	0.0856	5064.3	[114]
		WPU/MXene/NiFe_2_O_4_	2	64.7	8.2–12.4	0.0382	1694	[115]
		PLCNF/gelatin/MXene	2	62.9	8.2–12.4		14,230	[116]
		MXene/CNF	2	74.56	8.2–12.4	0.008	46,600	[117]
		MXene/CMC	1.3	52.15	8.2–12.4	0.0282	11,344.36	[118]
		MXene/CNF/APP	8	55	8.2–12.4	0.012	5729	[119]
		C-MXene/PAM/SA	1	26.8	8.2–12.4			[120]
	Hydrogel	MXene sediment/PVA	9	90	8.2–12.4			[121]
		MXene/PAA	0.13	23.2	200–2000			[122]
		MXene/PVA	2	57	8.2–12.4			[60]
		MXene/PVA/Gly	1	33.6	8.2–12.4	0.442		[123]
		MXene/CNF	5	30	8.2–12.4	0.0149		[61]
		MXene	0.045	92	8.2–12.4	2.39	8554	[42]
Film		MXene/SA	0.009	57	8.2–12.4	2.11	30,830	[42]
		MXene/CNF	0.047	24	8.2–12.4	1.92	2647	[67]
		MXene/ANF	0.017	33	8.2–12.4	1.25	15,529	[68]
		MXene/HEC	0.1	24	8.2–12.4	0.34	7000	[69]
		MXene/PEDOT: PSS	0.011	42.1	8.2–12.4	1.96	19,497	[71]
		MXene/PANI	0.04	36	8.2–12.4			[72]
		MXene/CNT	0.0002	29	8.2–12.4	2.49	58,187	[80]
		MXene/PVA	0.027	44.4	8.2–12.4	1.74	9643	[81]
		MXene/AgNW	0.012	54	8.2–12.4			[89]
		MXene/cellulose	0.2	43	8.2–18	0.49		[88]
		MXene/CF	0.33	36	8.2–12.4		0.78	[87]
		MXene/CNT/CNF	0.038	38.4	8.2–12.4	1.26	8020	[94]
		MXene/CNF/silver	0.046	50.7	8.2–12.4		≈9000	[95]
		MXene/PVDF/Ni	0.36	34.4	8.2–12.4		1177	[96]
		MXene/TiO_2_/rGO	0. 00907	28	2–18		30,293	[97]
		MXene/GO	0.012	53.5	8.2–12.4		7167	[98]
		MXene/AgNW/NC	0.024	42	8.2–12.4	1.5	16,724	[99]
		MXene/Al^3+^	0.039	80	8.2–12.4			[124]
Segregated structure		MXene/PS	2	62	8.2–12.4	1.21	255.2	[100]

Owing to its superior metallic conductivity, MXenes provide state-of-the-art EMI shielding performances, surpassing numerous other conducting materials and their composites at equivalent thicknesses. MXene systems face several obstacles that must be addressed in the future: (1) although synthesis techniques for more than 30 distinct MXenes have been described, only a few, including Ti_3_C_2_, Ti_2_C, Mo_2_TiC_2_, Mo_2_TiC_3_, and Ti_3_CN, have been studied. To elucidate the roles of basic composition, layer construction, and transition metal arrangement in the EMI shielding nature of MXenes, it is necessary to investigate other MXenes. It is also necessary to explore an efficient, green and scalable MXene preparation method for industrialization. (2) Typically, highly conductive MXenes contribute substantially to EMI shielding, which may result in secondary pollution. Porous foams, aerogels, and segregated constructions have been proposed to address these issues. Nevertheless, the generation of MXene-based shielding materials with varied structural factors, such as metastructures, remains necessary to significantly increase the absorption of incoming EMWs. (3) Owing to the hydrophilic properties of MXenes, MXene/polymer composites and hybrids with other nanomaterials can only be fabricated under aqueous conditions. To expand the structural design scope, MXenes shall be synthesized and investigated with hydrophobic surfaces and appropriate organic dispersants.

Moreover, MXenes have significant potential for further investigation into its structure, properties, and integration with other materials, aiming to enhance its EMI shielding capabilities and broaden its use in related fields. Additional investigation is required to optimize the inherent properties and explore possible applications in many domains. Finally, although the reported MXene-based macrostructures exhibit satisfactory EMI shielding performances, research on MXenes is still in its infancy. Thus, there is crucial range for the development of new MXenes and investigating their shielding capabilities. We believe that subsequent research can overcome the various difficulties to achieve all-encompassing performance improvements and hope that this comprehensive assessment will provide new insights and guidelines for academia to propel the growth of next-generation EMI shielding materials.

## References

[1] Gholamirad F, Ge JQ, Sadati M, Wang GA, Taheri-Qazvini N (2022). Tuning the self-assembled morphology of Ti_3_C_2_T_*x*_ MXene-based hybrids for high-performance electromagnetic interference shielding. ACS Appl. Mater. Interfaces.

[2] Shen X, Kim JK (2022). Graphene and MXene-based porous structures for multifunctional electromagnetic interference shielding. Nano Res..

[3] Wu FS, Tian ZH, Hu PY, Tang JW, Xu XQ, Pan L, Liu J, Zhang PG, Sun ZM (2022). Lightweight and flexible PAN@PPy/MXene films with outstanding electromagnetic interference shielding and joule heating performance. Nanoscale.

[4] Ali A, Hussain F, Tahir MF, Ali M, Khan MZ, Tomkova B, Militky J, Noman MT, Azeem M (2022). Fabrication of conductive, high strength and electromagnetic interference (EMI) shielded green composites based on waste materials. Polymers-Basel.

[5] Zhou ZH, Song QC, Huang BX, Feng SY, Lu CH (2021). Facile fabrication of densely packed Ti_3_C_2_ MXene/nanocellulose composite films for enhancing electromagnetic interference shielding and electro-/photothermal performance. ACS Nano.

[6] Feng SY, Yi Y, Chen BX, Deng PC, Zhou ZH, Lu CH (2022). Rheology-guided assembly of a highly aligned MXene/cellulose nanofiber composite film for high-performance electromagnetic interference shielding and infrared stealth. ACS Appl. Mater. Interfaces.

[7] Chen YS, Huang Y, Park CB, Che RC, Yu ZZ (2022). Electromagnetic interference shielding and microwave absorption materials: a virtual special issue. Carbon.

[8] Cheng JY, Li CB, Xiong YF, Zhang HB, Raza H, Ullah S, Wu JY, Zheng GP, Cao Q, Zhang DQ, Zheng QB, Che RC (2022). Recent advances in design strategies and multifunctionality of flexible electromagnetic interference shielding materials. Nano-Micro Lett..

[9] Cheng ML, Ying MF, Zhao RZ, Ji LZ, Li HX, Liu XG, Zhang J, Li YX, Dong XL, Zhang XF (2022). Transparent and flexible electromagnetic interference shielding materials by constructing sandwich AgNW@MXene/wood composites. ACS Nano.

[10] Gao YY, Bao D, Zhang MH, Cui YX, Xu F, Shen XS, Zhu YJ, Wang HY (2022). Millefeuille-inspired thermal interface materials based on double self-assembly technique for efficient microelectronic cooling and electromagnetic interference shielding. Small.

[11] Song JW, Xu KJ, He J, Ye HJ, Xu LX (2023). Three-dimensional graphene/carbon nanotube electromagnetic shielding composite material based on melamine resin foam template. Polym. Compos..

[12] Asandulesa M, Hamciuc C, Pui A, Virlan C, Lisa G, Barzic AI, Oprisan B (2023). Cobalt ferrite/polyetherimide composites as thermally stable materials for electromagnetic interference shielding uses. Int. J. Mol. Sci..

[13] Zhang TY, Hu H, Wang JL, Fen Z, Guo JW, Liu XL (2022). Research on electromagnetic shielding effectiveness of multi-layered LZ91/Al alloy composite materials by asynchronous accumulative roll bonding. Mater. Sci..

[14] Lalan V, Ganesanpotti S (2022). The smallest anions, induced porosity and graphene interfaces in C12A7: e^–^ electrides: a paradigm shift in electromagnetic absorbers and shielding materials. J. Mater. Chem. C.

[15] Lv QN, Tao XY, Shi SH, Li YJ, Chen N (2022). From materials to components: 3D-printed architected honeycombs toward high-performance and tunable electromagnetic interference shielding. Compos. B Eng..

[16] Feng SY, Zhan ZY, Yi Y, Zhou ZH, Lu CH (2022). Facile fabrication of MXene/cellulose fiber composite film with homogeneous and aligned structure via wet co-milling for enhancing electromagnetic interference shielding performance. Compos. Part A—Appl. S..

[17] Rajan A, Solaman SK, Ganesanpotti S (2023). Design and fabrication of layered electromagnetic interference shielding materials: a cost-effective strategy for performance prediction and efficiency tuning. ACS Appl. Mater. Interfaces.

[18] Kumar R, Maji BC, Krishnan M (2020). Synthesis of 2D material MXene from Ti_3_AlC_2_ max-phase for electromagnetic shielding applications. AIP Conf. Proc..

[19] Yang YF, Wu N, Li B, Liu W, Pan F, Zeng ZH, Liu JR (2022). Biomimetic porous MXene sediment-based hydrogel for high-performance and multifunctional electromagnetic interference shielding. ACS Nano.

[20] Li XL, Yin XW, Liang S, Li MH, Cheng LF, Zhang LT (2019). 2D carbide MXene Ti_2_CT_*x*_ as a novel high-performance electromagnetic interference shielding material. Carbon.

[21] Gogotsi Y, Simon P (2011). True performance metrics in electrochemical energy storage. Science.

[22] Liu HB, Fu RL, Su XQ, Wu BY, Wang H, Xu Y, Liu XH (2021). MXene confined in shape-stabilized phase change material combining enhanced electromagnetic interference shielding and thermal management capability. Compos. Sci. Technol..

[23] Wang JF, Kang H, Cheng ZJ, Xie ZM, Wang YS, Liu YY, Fan ZM (2021). Research progress in Ti_3_C_2_T_x_ MXene-based electromagnetic interference shielding material. Nanoscale.

[24] Wang SJ, Li DS, Jiang L, Fang DN (2022). Flexible and mechanically strong MXene/FeCo@C decorated carbon cloth: a multifunctional electromagnetic interference shielding material. Compos. Sci. Technol..

[25] Sun XW, Wu XD, Deng PC, Tian D, Song YY, Zhao JQ, Li QY, Feng SY, Zhang J, Lu CH, Zou HW, Zhou ZH (2023). Facile and universal fabrication of cellulose nanofibers from bulk lignocellulose materials and their applications in multifunctional epidermal electrophysiological signals monitoring. Ind. Crop Prod..

[26] Thomassin J-M, Jérôme C, Pardoen T, Bailly C, Huynen I, Detrembleur C (2013). Polymer/carbon-based composites as electromagnetic interference (EMI) shielding materials. Mater. Sci. Eng. R.

[27] Schelkunoff SA (1943). A mathematical theory of linear arrays. Bell Syst. Tech. J..

[28] Y. Yi, S. Y. Feng, Z. H. Zhou, C. H. Lu. Wet mechanical grinding regulates the micro-nano interfaces and structure of MXene/PVA composite for enhanced mechanical properties and thermal conductivity. Compos. Part A—Appl. S. (2022). 10.1016/j.compositesa.2022.107232

[29] Nazir A, Yu HJ, Wang L, Haroon M, Ullah RS, Fahad S, Naveed KUR, Elshaarani T, Khan A, Usman M (2018). Recent progress in the modification of carbon materials and their application in composites for electromagnetic interference shielding. J. Mater. Sci..

[30] Iqbal A, Sambyal P, Koo CM (2020). 2D MXenes for electromagnetic shielding: a review. Adv. Funct. Mater..

[31] Wang C, Murugadoss V, Kong J, He Z, Mai X, Shao Q, Chen Y, Guo L, Liu C, Angaiah S, Guo Z (2018). Overview of carbon nanostructures and nanocomposites for electromagnetic wave shielding. Carbon.

[32] Zhang DQ, Liang S, Chai JX, Liu TT, Yang XY, Wang H, Cheng JY, Zheng GP, Cao MS (2019). Highly effective shielding of electromagnetic waves in MoS_2_ nanosheets synthesized by a hydrothermal method. J. Phys. Chem. Solids.

[33] Geetha S, Satheesh Kumar KK, Rao CRK, Vijayan M, Trivedi DC (2009). EMI shielding: methods and materials—a review. J. Appl. Polym. Sci..

[34] Kumar P, Narayan Maiti U, Sikdar A, Kumar Das T, Kumar A, Sudarsan V (2019). Recent advances in polymer and polymer composites for electromagnetic interference shielding: Review and future prospects. Polym. Rev..

[35] Schulz RB, Plantz VC, Brush DR (1988). Shielding theory and practice. IEEE T. Electromagn. C..

[36] Sankaran S, Deshmukh K, Ahamed MB, Pasha SKK (2018). Recent advances in electromagnetic interference shielding properties of metal and carbon filler reinforced flexible polymer composites: a review. Compos. Part A—Appl. Sci. Manuf..

[37] Wang HS, Wang GB, Li WL, Wang QT, Wei W, Jiang ZH, Zhang SL (2012). A material with high electromagnetic radiation shielding effectiveness fabricated using multi-walled carbon nanotubes wrapped with poly (ether sulfone) in a poly (ether ether ketone) matrix. J. Mater. Chem. C.

[38] Li CF, Zhou CX, Lv JB, Liang B, Li RK, Liu Y, Hu JH, Zeng K, Yang G (2019). Bio-molecule adenine building block effectively enhances electromagnetic interference shielding performance of polyimide-derived carbon foam. Carbon.

[39] Yuan Y, Yin WL, Yang ML, Xu F, Zhao X, Li JJ, Peng QY, He XD, Du SY, Li YB (2018). Lightweight, flexible and strong core-shell non-woven fabrics covered by reduced graphene oxide for high-performance electromagnetic interference shielding. Carbon.

[40] Yevick D, Friese T, Schmidt F (2001). A comparison of transparent boundary conditions for the fresnel equation. J. Comput. Chem..

[41] Zhang DQ, Cheng JY, Yang XY, Zhao B, Cao MS (2014). Electromagnetic and microwave absorbing properties of magnetite nanoparticles decorated carbon nanotubes/polyaniline multiphase heterostructures. J. Mater. Sci..

[42] Shahzad F, Alhabeb M, Hatter CB, Anasori B, Hong SM, Koo CM, Gogotsi Y (2016). Electromagnetic interference shielding with 2D transition metal carbides (MXenes). Science.

[43] Kumar R, Choudhary HK, Pawar SP, Bose S, Sahoo B (2017). Carbon encapsulated nanoscale iron/iron-carbide/graphite particles for EMI shielding and microwave absorption. Phys. Chem. Chem. Phys..

[44] Yun T, Kim H, Iqbal A, Cho YS, Lee GS, Kim MK, Kim SJ, Kim D, Gogotsi Y, Kim SO, Koo CM (2020). Electromagnetic shielding of monolayer MXene assemblies. Adv. Mater..

[45] Song Q, Ye F, Yin XW, Li W, Li HJ, Liu YS, Li KZ, Xie KY, Li XH, Fu QG, Cheng LF, Zhang LT, Wei BQ (2017). Carbon nanotube-multilayered graphene edge plane core-shell hybrid foams for ultrahigh-performance electromagnetic-interference shielding. Adv. Mater..

[46] Li Y, Tian X, Gao SP, Jing L, Li KR, Yang HT, Fu FF, Lee JY, Guo YX, Ho JS, Chen PY (2020). Reversible crumpling of 2D titanium carbide (MXene) nanocoatings for stretchable electromagnetic shielding and wearable wireless communication. Adv. Funct. Mater..

[47] Singh BP, Saini P, Gupta T, Garg P, Kumar G, Pande I, Pande S, Seth RK, Dhawan SK, Mathur RB (2011). Designing of multiwalled carbon nanotubes reinforced low density polyethylene nanocomposites for suppression of electromagnetic radiation. J. Nanopart. Res..

[48] Kim BR, Lee HK, Kim E, Lee SH (2010). Intrinsic electromagnetic radiation shielding/absorbing characteristics of polyaniline-coated transparent thin films. Synth. Met..

[49] M.Y. Peng, F.X. Qin. Clarification of basic concepts for electromagnetic interference shielding effectiveness. J. Appl. Phys. (2021). 10.1063/5.0075019

[50] Li YZ, Wang BJ, Sui XF, Xu H, Zhang LP, Zhong Y, Mao ZP (2017). Facile synthesis of microfibrillated cellulose/organosilicon/polydopamine composite sponges with flame retardant properties. Cellulose.

[51] Wang B, Li WF, Deng JP (2017). Chiral 3D porous hybrid foams constructed by graphene and helically substituted polyacetylene: preparation and application in enantioselective crystallization. J. Mater. Sci..

[52] Iqbal A, Shahzad F, Hantanasirisakul K, Kim MK, Kwon J, Hong J, Kim H, Kim D, Gogotsi Y, Koo CM (2020). Anomalous absorption of electromagnetic waves by 2D transition metal carbonitride Ti_3_C_n_T_*x*_ (MXene). Science.

[53] Hantanasirisakul K, Alhabeb M, Lipatov A, Maleski K, Anasori B, Salles P, Ieosakulrat C, Pakawatpanurut P, Sinitskii A, May SJ, Gogotsi Y (2019). Effects of synthesis and processing on optoelectronic properties of titanium carbonitride MXene. Chem. Mater..

[54] Cao MS, Cai YZ, He P, Shu JC, Cao WQ, Yuan J (2019). 2D MXenes: electromagnetic property for microwave absorption and electromagnetic interference shielding. Chem. Eng. J..

[55] Qian KP, Zhou QF, Wu HM, Fang JH, Miao M, Yang YH, Cao SM, Shi LY, Feng X (2021). Carbonized cellulose microsphere@void@MXene composite films with egg-box structure for electromagnetic interference shielding. Compos. Part. A—Appl. Sci. Manuf..

[56] Liu J, Zhang HB, Sun RH, Liu YF, Liu ZS, Zhou AG, Hydrophobic ZZYu (2017). flexible, and lightweight MXene foams for high-performance electromagnetic-interference shielding. Adv. Mater..

[57] Wu XY, Han BY, Zhang HB, Xie X, Tu TX, Zhang Y, Dai Y, Yang R, Compressible ZZYu (2020). durable and conductive polydimethylsiloxane-coated MXene foams for high-performance electromagnetic interference shielding. Chem. Eng. J..

[58] Zhao S, Zhang HB, Luo JQ, Wang QW, Xu B, Hong S, Yu ZZ (2018). Highly electrically conductive three-dimensional Ti_3_C_2_T_x_ MXene/reduced graphene oxide hybrid aerogels with excellent electromagnetic interference shielding performances. ACS Nano.

[59] Bian RJ, He GL, Zhi WQ, Xiang SL, Wang TW, Cai DY (2019). Ultralight MXene-based aerogels with high electromagnetic interference shielding performance. J. Mater. Chem. C.

[60] Yang YF, Li B, Wu N, Liu W, Zhao SY, Zhang CJ, Liu JR, Zeng ZH (2022). Biomimetic porous MXene-based hydrogel for high-performance and multifunctional electromagnetic interference shielding. ACS Mater. Lett..

[61] Bai Y, Bi SH, Wang WK, Ding N, Lu YY, Jiang MY, Ding CB, Zhao WW, Liu N, Bian J, Liu SJ, Zhao Q (2022). Biocompatible, stretchable, and compressible cellulose/MXene hydrogel for strain sensor and electromagnetic interference shielding. Soft Mater..

[62] Hu FY, Wang XH, Bao S, Song LM, Zhang S, Niu HH, Fan BB, Zhang R, Li HX (2022). Tailoring electromagnetic responses of delaminated Mo_2_TiC_2_T_*x*_ MXene through the decoration of Ni particles of different morphologies. Chem. Eng. J..

[63] Khaledialidusti R, Mishra AK, Barnoush A (2020). Atomic defects in monolayer ordered double transition metal carbide (Mo_2_TiC_2_T_*x*_) MXene and CO_2_ adsorption. J. Mater. Chem. C.

[64] Wang JY, He PL, Shen YL, Dai LX, Li Z, Wu Y, An CH (2021). FeNi nanoparticles on Mo_2_TiC_2_T_*x*_ MXene@nickel foam as robust electrocatalysts for overall water splitting. Nano Res..

[65] X.Y. Zhao, K.W. Tang, C. Lee, C.F. Du, H. Yu, X.M. Wang, W.H. Qi, Q. Ye, Q. Y. Yan. Promoting the water-reduction kinetics and alkali tolerance of MoNi_4_ nanocrystals via a Mo_2_TiC_2_T_*x*_ induced built-in electric field. Small (2022). 10.1002/smll.202107541 10.1002/smll.202107541 35254002

[66] He P, Wang XX, Cai YZ, Shu JC, Zhao QL, Yuan J, Cao MS (2019). Tailoring Ti_3_C_2_T_x_ nanosheets to tune local conductive network as an environmentally friendly material for highly efficient electromagnetic interference shielding. Nanoscale.

[67] Cao WT, Chen FF, Zhu YJ, Zhang YG, Jiang YY, Ma MG, Chen F (2018). Binary strengthening and toughening of MXene/cellulose nanofiber composite paper with nacre-inspired structure and superior electromagnetic interference shielding properties. ACS Nano.

[68] Xie F, Jia FF, Zhuo LH, Lu ZQ, Si LM, Huang JZ, Zhang MY, Ma Q (2019). Ultrathin MXene/aramid nanofiber composite paper with excellent mechanical properties for efficient electromagnetic interference shielding. Nanoscale.

[69] He P, Cao MS, Cai YZ, Shu JC, Cao WQ, Yuan J (2020). Self-assembling flexible 2D carbide MXene film with tunable integrated electron migration and group relaxation toward energy storage and green EMI shielding. Carbon.

[70] Wang L, Chen LX, Song P, Liang CB, Lu YJ, Qiu H, Zhang YL, Kong J, Gu JW (2019). Fabrication on the annealed Ti_3_C_2_T_x_ MXene/epoxy nanocomposites for electromagnetic interference shielding application. Compos. B Eng..

[71] Liu RT, Miao M, Li YH, Zhang JF, Cao SM, Feng X (2018). Ultrathin biomimetic polymeric Ti_3_C_2_T_*x*_ MXene composite films for electromagnetic interference shielding. ACS Appl. Mater. Interfaces.

[72] Zhang YL, Wang L, Zhang JL, Song P, Xiao ZR, Liang CB, Qiu H, Kong J, Gu JW (2019). Fabrication and investigation on the ultra-thin and flexible Ti_3_C_2_T_*x*_/Co-doped polyaniline electromagnetic interference shielding composite films. Compos. Sci. Technol..

[73] Yang FS, Li CL, Xu WZ, Cai ZS (2019). Multifunctional antifogging coatings based on ZrO_2_ and SiO_2_ nanoparticles by spray-spin-blow layer-by-layer assembly. J. Mater. Res..

[74] Zhao YB, Liu HP, Li CY, Chen Y, Li SQ, Zeng RC, Wang ZL (2018). Corrosion resistance and adhesion strength of a spin-assisted layer-by-layer assembled coating on AZ31 magnesium alloy. Appl. Surf. Sci..

[75] Javaid S, Mahmood A, Nasir H, Iqbal M, Ahmed N, Ahmad NM (2022). Layer-by-layer self-assembled dip coating for antifouling functionalized finishing of cotton textile. Polymers.

[76] Alongi J, Carosio F, Frache A, Malucelli G (2013). Layer by layer coatings assembled through dipping, vertical or horizontal spray for cotton flame retardancy. Carbohydr. Polym..

[77] Xiong MM, Ren ZH, Liu WJ (2019). Fabrication of uv-resistant and superhydrophobic surface on cotton fabric by functionalized polyethyleneimine/SiO_2_ via layer-by-layer assembly and dip-coating. Cellulose.

[78] Charinpanitkul T, Suthabanditpong W, Watanabe H, Shirai T, Faungnawakij K, Viriya-empikul N, Fuji M (2012). Improved hydrophilicity of zinc oxide-incorporated layer-by-layer polyelectrolyte film fabricated by dip coating method. J. Ind. Eng. Chem..

[79] Li B, Wu N, Yang YF, Pan F, Wang CX, Wang G, Xiao L, Liu W, Liu JR, Zeng ZH (2022). Graphene oxide-assisted multiple cross-linking of MXene for large-area, high-strength, oxidation-resistant, and multifunctional films. Adv. Funct. Mater..

[80] Weng GM, Li JY, Alhabeb M, Karpovich C, Wang H, Lipton J, Maleski K, Kong J, Shaulsky E, Elimelech M, Gogotsi Y, Taylor AD (2018). Layer-by-layer assembly of cross-functional semi-transparent MXene-carbon nanotubes composite films for next-generation electromagnetic interference shielding. Adv. Funct. Mater..

[81] Jin XX, Wang JF, Dai LZ, Liu XY, Li L, Yang YY, Cao YX, Wang WJ, Wu H, Guo SY (2020). Flame-retardant poly(vinyl alcohol)/MXene multilayered films with outstanding electromagnetic interference shielding and thermal conductive performances. Chem. Eng. J..

[82] Zhou B, Zhang Z, Li YL, Han GJ, Feng YZ, Wang B, Zhang DB, Ma JM, Liu CT (2020). Flexible, robust, and multifunctional electromagnetic interference shielding film with alternating cellulose nanofiber and MXene layers. ACS Appl. Mater. Interfaces.

[83] Wang Y, Wang W, Qi QB, Xu N, Yu D (2020). Layer-by-layer assembly of pdms-coated nickel ferrite/multiwalled carbon nanotubes/cotton fabrics for robust and durable electromagnetic interference shielding. Cellulose.

[84] Jia XC, Shen B, Zhang LH, Zheng WG (2020). Waterproof MXene-decorated wood-pulp fabrics for high-efficiency electromagnetic interference shielding and joule heating. Compos. B Eng..

[85] Wang XF, Lei ZW, Ma XD, He GF, Xu T, Tan J, Wang LL, Zhang XS, Qu LJ, Zhang XJ (2022). A lightweight MXene-coated nonwoven fabric with excellent flame retardancy, EMI shielding, and electrothermal/photothermal conversion for wearable heater. Chem. Eng. J..

[86] Zhang HY, Chen JY, Ji H, Wang N, Feng S, Xiao H (2021). Electromagnetic interference shielding with absorption-dominant performance of Ti_3_C_2_T_x_ MXene/non-woven laminated fabrics. Text. Res. J..

[87] Zhang XS, Wang XF, Lei ZW, Wang LL, Tian MW, Zhu SF, Xiao H, Tang XN, Qu LJ (2020). Flexible MXene-decorated fabric with interwoven conductive networks for integrated joule heating, electromagnetic interference shielding, and strain sensing performances. Acs. Appl. Mater. Interfaces.

[88] Liu LX, Chen W, Zhang HB, Wang QW, Guan FL, Yu ZZ (2019). Flexible and multifunctional silk textiles with biomimetic leaf-like MXene/silver nanowire nanostructures for electromagnetic interference shielding, humidity monitoring, and self-derived hydrophobicity. Adv. Funct. Mater..

[89] Hu DW, Huang XY, Li ST, Jiang PK (2020). Flexible and durable cellulose/MXene nanocomposite paper for efficient electromagnetic interference shielding. Compos. Sci. Technol..

[90] Ma C, Liu T, Xin W, Xi GQ, Ma MG (2019). Breathable and wearable MXene-decorated air-laid paper with superior folding endurance and electromagnetic interference-shielding performances. Front. Mater..

[91] Fan ZM, Wang DL, Yuan Y, Wang YS, Cheng ZJ, Liu YY, Xie ZM (2020). A lightweight and conductive MXene/graphene hybrid foam for superior electromagnetic interference shielding. Chem. Eng. J..

[92] Sang M, Wu YX, Liu S, Bai LF, Wang S, Jiang WQ, Gong XL, Xuan SH (2021). Flexible and lightweight melamine sponge/MXene/polyborosiloxane (MSMP) hybrid structure for high-performance electromagnetic interference shielding and anti-impact safe-guarding. Compos. B Eng..

[93] Li B, Yang YF, Wu N, Zhao SY, Jin H, Wang GL, Li XY, Liu W, Liu JR, Zeng ZH (2022). Bicontinuous, high-strength, and multifunctional chemical-cross-linked MXene/superaligned carbon nanotube film. ACS Nano.

[94] Cao WT, Ma C, Tan S, Ma MG, Wan PB, Chen F (2019). Ultrathin and flexible CNTs/MXene/cellulose nanofibrils composite paper for electromagnetic interference shielding. Nano-Micro. Lett..

[95] Xin W, Xi GQ, Cao WT, Ma C, Liu T, Ma MG, Bian J (2019). Lightweight and flexible MXene/CNF/silver composite membranes with a brick-like structure and high-performance electromagnetic-interference shielding. Rsc. Adv..

[96] Wang SJ, Li DS, Jiang L (2019). Synergistic effects between MXenes and Ni chains in flexible and ultrathin electromagnetic interference shielding films. Adv. Mater. Interfaces.

[97] Xiang C, Guo RH, Lin SJ, Jiang SX, Lan JW, Wang C, Cui C, Xiao HY, Zhang Y (2019). Lightweight and ultrathin TiO_2_-Ti_3_C_2_T_x_/graphene film with electromagnetic interference shielding. Chem. Eng. J..

[98] Liu J, Liu ZS, Zhang HB, Chen W, Zhao ZF, Wang QW, Yu ZZ (2020). Ultrastrong and highly conductive MXene-based films for high-performance electromagnetic interference shielding. Adv. Electron. Mater..

[99] Miao M, Liu RT, Thaiboonrod S, Shi LY, Cao SM, Zhang JF, Fang JH, Feng X (2020). Silver nanowires intercalating Ti_3_C_2_T_x_ MXene composite films with excellent flexibility for electromagnetic interference shielding. J. Mater. Chem. C.

[100] Sun RH, Zhang HB, Liu J, Xie X, Yang R, Li Y, Hong S, Yu ZZ (2017). Highly conductive transition metal carbide/carbonitride(MXene)@polystyrene nanocomposites fabricated by electrostatic assembly for highly efficient electromagnetic interference shielding. Adv. Funct. Mater..

[101] Xu HL, Yin XW, Li XL, Li MH, Liang S, Zhang LT, Cheng LF (2019). Lightweight Ti_2_CT_x_ MXene/poly (vinyl alcohol) composite foams for electromagnetic wave shielding with absorption-dominated feature. ACS Appl. Mater. Interfaces.

[102] Qi FQ, Wang L, Zhang YL, Ma ZL, Qiu H, Gu JW (2021). Robust Ti_3_C_2_T_x_ MXene/starch derived carbon foam composites for superior EMI shielding and thermal insulation. Mater. Today Phys..

[103] Zeng ZH, Wu N, Wei JJ, Yang YF, Wu TT, Li B, Hauser SB, Yang WD, Liu JR, Zhao SY (2022). Porous and ultra-flexible crosslinked MXene/polyimide composites for multifunctional electromagnetic interference shielding. Nano-Micro. Lett..

[104] Xu ZJ, Ding X, Li SK, Huang FZ, Wang BJ, Wang SP, Zhang X, Liu FH, Zhang H (2022). Oxidation-resistant MXene-based melamine foam with ultralow-percolation thresholds for electromagnetic-infrared compatible shielding. ACS Appl. Mater. Interfaces.

[105] Lu ZQ, Jia FF, Zhuo LH, Ning DD, Gao K, Xie F (2021). Micro-porous MXene/aramid nanofibers hybrid aerogel with reversible compression and efficient EMI shielding performance. Compos. B Eng..

[106] Wu N, Yang YF, Wang CX, Wu QL, Pan F, Zhang RA, Liu JR, Zeng ZH (2023). Ultrathin cellulose nanofiber assisted ambient-pressure-dried, ultralight, mechanically robust, multifunctional MXene aerogels. Adv. Mater..

[107] Qi CZ, Wu XY, Liu J, Luo XJ, Zhang HB, Yu ZZ (2023). Highly conductive calcium ion-reinforced MXene/sodium alginate aerogel meshes by direct ink writing for electromagnetic interference shielding and joule heating. J. Mater. Sci. Technol..

[108] Yang GY, Wang SZ, Sun HT, Yao XM, Li CB, Li YJ, Jiang JJ (2021). Ultralight, conductive Ti_3_C_2_T_x_ MXene/PEDOT: PSS hybrid aerogels for electromagnetic interference shielding dominated by the absorption mechanism. ACS Appl. Mater. Interfaces.

[109] Du YQ, Xu J, Fang JY, Zhang YT, Liu XY, Zuo PY, Zhuang QX (2022). Ultralight, highly compressible, thermally stable MXene/aramid nanofiber anisotropic aerogels for electromagnetic interference shielding. J. Mater. Chem. A.

[110] Sambyal P, Iqbal A, Hong J, Kim H, Kim MK, Hong SM, Han MK, Gogotsi Y, Koo CM (2019). Ultralight and mechanically robust Ti_3_C_2_T_x_ hybrid aerogel reinforced by carbon nanotubes for electromagnetic interference shielding. ACS Appl. Mater. Interfaces.

[111] Weng CX, Wang GR, Dai ZH, Pei YM, Liu LQ, Zhang Z (2019). Buckled AgNW/MXene hybrid hierarchical sponges for high-performance electromagnetic interference shielding. Nanoscale.

[112] Wu SQ, Chen DM, Han WB, Xie YS, Zhao GD, Dong S, Tan MY, Huang H, Xu SB, Chen GQ, Cheng Y, Zhang XH (2022). Ultralight and hydrophobic MXene/chitosan-derived hybrid carbon aerogel with hierarchical pore structure for durable electromagnetic interference shielding and thermal insulation. Chem. Eng. J..

[113] Zhao DQ, Dang LY, Wang GG, Sun N, Deng XY, Han JC, Zhu JQ, Yang Y (2022). Multifunctional, superhydrophobic and highly elastic MXene/bacterial cellulose hybrid aerogels enabled via silylation. J Mater. Chem. A.

[114] Guo ZZ, Ren PG, Yang F, Wu T, Zhang LX, Chen ZY, Huang SQ, Ren F (2023). Mof-derived Co/C and MXene Co-decorated cellulose-derived hybrid carbon aerogel with a multi-interface architecture toward absorption-dominated ultra-efficient electromagnetic interference shielding. ACS Appl. Mater. Interfaces.

[115] Wang Y, Qi QB, Yin G, Wang W, Flexible DYu (2021). ultralight, and mechanically robust waterborne polyurethane/Ti_3_C_2_T_*x*_ MXene/nickel ferrite hybrid aerogels for high-performance electromagnetic interference shielding. ACS Appl. Mater. Interfaces.

[116] Li YH, Chen Y, He XF, Xiang ZY, Heinze T, Qi HS (2022). Lignocellulose nanofibril/gelatin/MXene composite aerogel with fire-warning properties for enhanced electromagnetic interference shielding performance. Chem. Eng. J..

[117] Zeng ZH, Wang CX, Siqueira G, Han DX, Huch A, Abdolhosseinzadeh S, Heier J, Nuesch F, Zhang CF, Nystrom G (2020). Nanocellulose-MXene biomimetic aerogels with orientation-tunable electromagnetic interference shielding performance. Adv. Sci..

[118] Cheng Y, Zhu WD, Lu XF, Wang C (2022). Lightweight and flexible MXene/carboxymethyl cellulose aerogel for electromagnetic shielding, energy harvest and self-powered sensing. Nano Energy.

[119] Zhang Y, Yu J, Lu JY, Zhu CJ, Qi DM (2021). Facile construction of 2D MXene (Ti_3_C_2_T_*x*_) based aerogels with effective fire-resistance and electromagnetic interference shielding performance. J. Alloys Compd..

[120] Xia BH, Li T, Chen MQ, Wang SB, Dong WF (2022). L-citrulline-modified Ti_3_C_2_T_*x*_ MXene nanosheets embedded in polyacrylamide/sodium alginate hydrogels for electromagnetic interference shielding. ACS Appl. Nano Mater..

[121] Zhao T, Xie PY, Wan HJ, Ding TP, Liu MQ, Xie JL, Li EE, Chen XQ, Wang TW, Zhang Q, Wei YY, Gong YB, Wen QY, Hu M, Qiu CW, Xiao X (2023). Ultrathin MXene assemblies approach the intrinsic absorption limit in the 0.5–10 THz band. Nat. Photonics.

[122] Zhu YY, Liu J, Guo T, Wang JJ, Tang XZ, Nicolosi V (2021). Multifunctional Ti_3_C_2_T_x_ MXene composite hydrogels with strain sensitivity toward absorption-dominated electromagnetic- interference shielding. ACS Nano.

[123] Yu YH, Yi P, Xu WB, Sun X, Deng G, Liu XF, Shui JL, Yu RH (2022). Environmentally tough and stretchable MXene organohydrogel with exceptionally enhanced electromagnetic interference shielding performances. Nano-Micro. Lett..

[124] Liu ZS, Zhang Y, Zhang HB, Dai Y, Liu J, Li XF, Yu ZZ (2020). Electrically conductive aluminum ion-reinforced MXene films for efficient electromagnetic interference shielding. J. Mater. Chem. C.

[125] Liu N, Li QQ, Wan HJ, Chang LB, Wang H, Fang JH, Ding TP, Wen QY, Zhou LJ, Xiao X (2022). High-temperature stability in air of Ti_3_C_2_T_x_ MXene-based composite with extracted bentonite. Nat. Commun..

[126] Zhang TZ, Chang LB, Zhang XF, Wan HJ, Liu N, Zhou LJ, Xiao X (2022). Simultaneously tuning interlayer spacing and termination of MXenes by Lewis-basic halides. Nat. Commun..

[127] Xu DJ, Huang Q, Yang LK, Chen YJ, Lu ZM, Liu HJ, Han PJ, Guo L, Wang C, Liu CC (2023). Experimental design of composite films with thermal management and electromagnetic shielding properties based on polyethylene glycol and MXene. Carbon.

[128] Xue TT, Yang Y, Yu DY, Wali Q, Wang ZY, Cao XS, Fan W, Liu TX (2023). 3D printed integrated gradient-conductive MXene/CNT/polyimide aerogel frames for electromagnetic interference shielding with ultra-low reflection. Nano-Micro. Lett..

[129] Zhu M, Yan XX, Xu HL, Xu YJ, Kong L (2021). Ultralight, compressible, and anisotropic MXene@wood nanocomposite aerogel with excellent electromagnetic wave shielding and absorbing properties at different directions. Carbon.

[130] Yang YF, Han MR, Liu W, Wu N, Liu JR (2022). Hydrogel-based composites beyond the porous architectures for electromagnetic interference shielding. Nano Res..

[131] Wang Z, Cheng Z, Xie L, Hou XL, Fang CQ (2021). Flexible and lightweight Ti_3_C_2_T_x_ MXene/Fe_3_O_4_@PANI composite films for high-performance electromagnetic interference shielding. Ceram. Int..

